# Spontaneous atopic dermatitis is mediated by innate immunity, with the secondary lung inflammation of the atopic march requiring adaptive immunity

**DOI:** 10.1016/j.jaci.2015.06.045

**Published:** 2016-02

**Authors:** Sean P. Saunders, Tara Moran, Achilleas Floudas, Felicity Wurlod, Agnieszka Kaszlikowska, Maryam Salimi, Emma M. Quinn, Christopher J. Oliphant, Gabriel Núñez, Ross McManus, Emily Hams, Alan D. Irvine, Andrew N.J. McKenzie, Graham S. Ogg, Padraic G. Fallon

**Affiliations:** a Trinity Biomedical Sciences Institute, Trinity College Dublin, Dublin, Ireland; b National Children's Research Centre, Our Lady's Children's Hospital, Dublin, Ireland; h Department of Paediatric Dermatology, Our Lady's Children's Hospital, Dublin, Ireland; c MRC Human Immunology Unit, NIHR Biomedical Research Centre, Radcliffe Department of Medicine, University of Oxford, Oxford, United Kingdom; d Institute of Molecular Medicine, St James's Hospital, Dublin, Ireland; e MRC Laboratory of Molecular Biology, Cambridge, United Kingdom; f Biosceptre, Babraham Research Campus, Babraham, Cambridge, United Kingdom; g Department of Pathology and Comprehensive Cancer Center, University of Michigan, Ann Arbor, Mich

**Keywords:** Allergy, asthma, atopic dermatitis, atopy, eczema, filaggrin, flaky tail, type 2 innate lymphoid cells, innate immunity, mouse, mutation, AD, Atopic dermatitis, AHR, Airway hyperresponsiveness, C_dyn_, Dynamic lung compliance, CFP, Cerulean fluorescent protein, dLN, Draining lymph node, eGFP, Enhanced green fluorescent protein, *FLG*, Human filaggrin gene, *Flg*, Murine filaggrin gene, iILC2, Inflammatory type 2 innate lymphoid cells, ILC2, Type 2 innate lymphoid cell, IL-7Rα, IL-7 receptor α, NBNT, Non-B/non-T cell, NF-κB, Nuclear factor κB, nILC2, Natural type 2 innate lymphoid cell, R_L_, Lung resistance, ROR, Retinoic acid–related orphan receptor, TSLP, Thymic stromal lymphopoietin, WT, Wild-type

## Abstract

**Background:**

Atopic dermatitis (AD) is an inflammatory skin condition that can occur in early life, predisposing to asthma development in a phenomenon known as the atopic march. Although genetic and environmental factors are known to contribute to AD and asthma, the mechanisms underlying the atopic march remain poorly understood. Filaggrin loss-of-function mutations are a major genetic predisposer for the development of AD and progression to AD-associated asthma.

**Objective:**

We sought to experimentally address whether filaggrin mutations in mice lead to the development of spontaneous eczematous inflammation and address the aberrant immunologic milieu arising in a mouse model of filaggrin deficiency.

**Methods:**

Filaggrin mutant mice were generated on the proallergic BALB/c background, creating a novel model for the assessment of spontaneous AD-like inflammation. Independently recruited AD case collections were analyzed to define associations between filaggrin mutations and immunologic phenotypes.

**Results:**

Filaggrin-deficient mice on a BALB/c background had profound spontaneous AD-like inflammation with progression to compromised pulmonary function with age, reflecting the atopic march in patients with AD. Strikingly, skin inflammation occurs independently of adaptive immunity and is associated with cutaneous expansion of IL-5–producing type 2 innate lymphoid cells. Furthermore, subjects with filaggrin mutations have an increased frequency of type 2 innate lymphoid cells in the skin in comparison with control subjects.

**Conclusion:**

This study provides new insights into our understanding of the atopic march, with innate immunity initiating dermatitis and the adaptive immunity required for subsequent development of compromised lung function.

There has been a profound increase in the incidence of atopic disease morbidity in developed societies in recent decades. Atopic individuals, who are characterized by increased serum IgE levels, are predisposed to having allergies such as atopic dermatitis (AD) and asthma. AD is heritable and characterized by pruritic eczematous lesions, with approximately 20% of children affected in the developed world.^1^ The cause of AD is multifactorial, with interplay between genetic predisposition and environmental factors initiating aberrant inflammation.^2^ The term atopic march encapsulates the predisposition of patients with AD in infancy to progress to secondary allergic disorders, such as asthma.^3^ Although AD as the first manifestation of atopic diathesis in early life is well established, how AD development primes progression to secondary allergies is not known.

Loss-of-function mutations in the human filaggrin gene *(FLG)* have been identified as the major genetic predisposing factor for AD development,^4^, ^5^, ^6^ and in the context of the atopic march, patients with AD with *FLG* mutations are predisposed to the development of asthma.^7^, ^8^ We previously identified a mutation in the murine filaggrin gene *(Flg)* in the “flaky tail” double-mutant *(Matt*^*ma/ma*^*Flg*^*ft/ft*^*)* mouse strain, resulting in a lack of filaggrin protein in the skin.^9^ We recently separated the matted and filaggrin mutations present in *Matt*^*ma/ma*^*Flg*^*ft/ft*^ flaky tail mice.^10^ We now show that filaggrin-deficient mice, analogous to *FLG* mutations in human subjects, have spontaneous dermatitis, become atopic and progress to lung inflammation with age. By using a mouse with a mutation in a gene implicated in the atopic march in human subjects, the roles of innate versus adaptive immunity are shown in the initial development of dermatitis and progression to aberrant lung inflammation. Filaggrin-deficient mice on a BALB/c background have a spontaneous expansion of IL-5–producing type 2 innate lymphoid cells (ILC2s) into the skin, with an increase in skin ILC2 numbers also seen in patients with *FLG* mutations, reinforcing the role of innate immunity in the development of AD.

## Methods

### Mice

All mice were congenic BALB/c strain, with BALB/c mice used as wild-type (WT) control animals. The *Flg*^*ft*^ and *ma* mutations in flaky tail *(Matt*^*ma/ma*^*Flg*^*ft/ft*^*)* mice (Stock a/a ma ft/ma ft/JSun; JR#9078; Jackson Laboratories, Bar Harbor, Me) were separated, and the *Flg*^*ft*^ mutation was backcrossed to the congenic C57BL/6J background in accordance with previously published methods.^10^ *Flg*^*ft/ft*^ C57BL/6J congenic mice were subsequently backcrossed to the congenic BALB/c background, and these mice were used in this study. *Il4*^*KN2*^,^11^ *Il5CFP*, *Il13eGFP*,^12^ *Il17eGFP* (Biocytogen, Worcester, Mass), and *Rag1*-deficient (Jackson Laboratories) mice were crossed with *Flg*^*ft/ft*^ mice in house. Mice expressing the luciferase transgene under the control of a nuclear factor κB (NF-κB) promoter (*NF-κB-Luc*; Caliper Life Sciences, Hopkinton, Mass) were crossed to *Flg*^*ft/ft*^ mice.

Mice were housed in specific pathogen-free conditions, with irradiated diet and bedding and water *ad libitum*. All animal experiments were performed in compliance with the Irish Department of Health and Children regulations and approved by Trinity College Dublin's BioResources ethical review board.

### Clinical scoring

The severity of skin inflammation was clinically scored longitudinally by using a system based on the macroscopic diagnostic criteria described by Saunders et al^10^ and adapted from assessment of skin inflammation in the Nc/Nga mouse model.^13^ In brief, a scoring system (0, none; 1, mild; 2, moderate; and 3, severe) was applied to the signs of edema, erythema, scaling, and erosion. The total score for each mouse was calculated from the sum of individual scores for each parameter.

### Analysis of airway hyperresponsiveness

Lung function or airway hyperresponsiveness (AHR) was analyzed in 32-week-old mice by using an invasive method in which mice were tracheostomized and ventilated with whole-body plethysmography^14^ by using a pneumotachograph linked to a transducer (EMMS, Hants, United Kingdom). Changes in lung resistance (R_L_) and dynamic lung compliance (C_dyn_) in response to increasing doses of nebulized and inhaled methacholine (10, 30, 60, and 100 mg/mL; Sigma-Aldrich, St Louis, Mo) were recorded, as previously described.^9^, ^15^

### Flow cytometric and cytokine analyses of human suction blisters

Suction blistering was performed on patient donors after obtaining informed written consent, and sample use was given ethical approval from the NRES Committee South Central, United Kingdom. Patients with moderate-to-severe AD were recruited and genotyped for *FLG* mutations (see this article's Online Repository at www.jacionline.org).^5^ Patients with WT, heterozygous, and compound heterozygous *FLG* status were included in the study. Suction blister cups were applied to the skin of patients with a vacuum pressure of 200 to 400 mm Hg, as previously described.^16^ Blisters were formed within 60 to 90 minutes, and suction was then removed. Twenty-four hours later, fluid was aspirated with a 30-gauge needle. Fluids were centrifuged at 1500 rpm for 5 minutes at 4°C, and cell pellets were resuspended in RPMI 1640 supplemented with 10% human serum.

For surface staining, single-cell suspensions were prepared in flow cytometry buffer. Live/dead violet (Invitrogen, Carlsbad, Calif) was used to determine cell viability. Directly conjugated antibodies with fluorescein isothiocyanate, phycoerythrin, phycoerythrin–Texas Red, peridinin-chlorophyll-protein complex, peridinin-chlorophyll-protein complex–Cy5.5, PeCy7, V450, allophycocyanin, and allophycocyanin-Cy7 were used. Human cells were stained with the BioLegend (San Diego, Calif) mAbs CD4 (MEM-241), CD8 (RPA-T8), CD11b (DCIS1/18), CD45 (H130), CD56 (B159), FcεRI (AER-37 [CRA-1]), and IL-7 receptor α (IL-7Rα; A019D5); the BD Biosciences (San Jose, Calif) mAbs CD3 (SK7), CD19 (SJ25C1), and CD14 (MφP9); the Abcam (Cambridge, United Kingdom) mAb CD11c (BU15); the Miltenyi Biotec (Bergisch Gladbach, Germany) mAb chemoattractant receptor–homologous molecule expressed on T_H_2 lymphocytes (BM16); and the R&D Systems (Minneapolis, Minn) mAb CD123 (FAB301C). Cells were acquired by using FACSDiva (BD Biosciences) or Summit software (Beckman Coulter, High Wycombe, United Kingdom) on an LSRFortessa or CyAn flow Cytometer, respectively. Lineage gating included CD3, CD4, CD8, CD14, CD19, CD56, CD11c, CD11b, FcεRI, and CD123. ILC2s were defined as Lin^−^CD45^+^IL-7Rα^+^ chemoattractant receptor–homologous molecule expressed on T_H_2 lymphocytes positive. FlowJo (TreeStar, Ashland, Ore) and Summit software were used for further data analysis. Blister fluid was analyzed with the MAGPIX Multiplex Array (Luminex, Austin, Tex), according to the manufacturer's instructions. Quantification of ILC2s and IL-1β levels in patient samples was performed in a blinded manner.

### Statistical analyses

Data are expressed as means ± SEMs and analyzed by using 2-way ANOVA or the unpaired Student *t* tests (Prism 6; GraphPad Software, La Jolla, Calif).

## Results

### Filaggrin deficiency leads to spontaneous dermatitis and atopy

**Fig 1:**
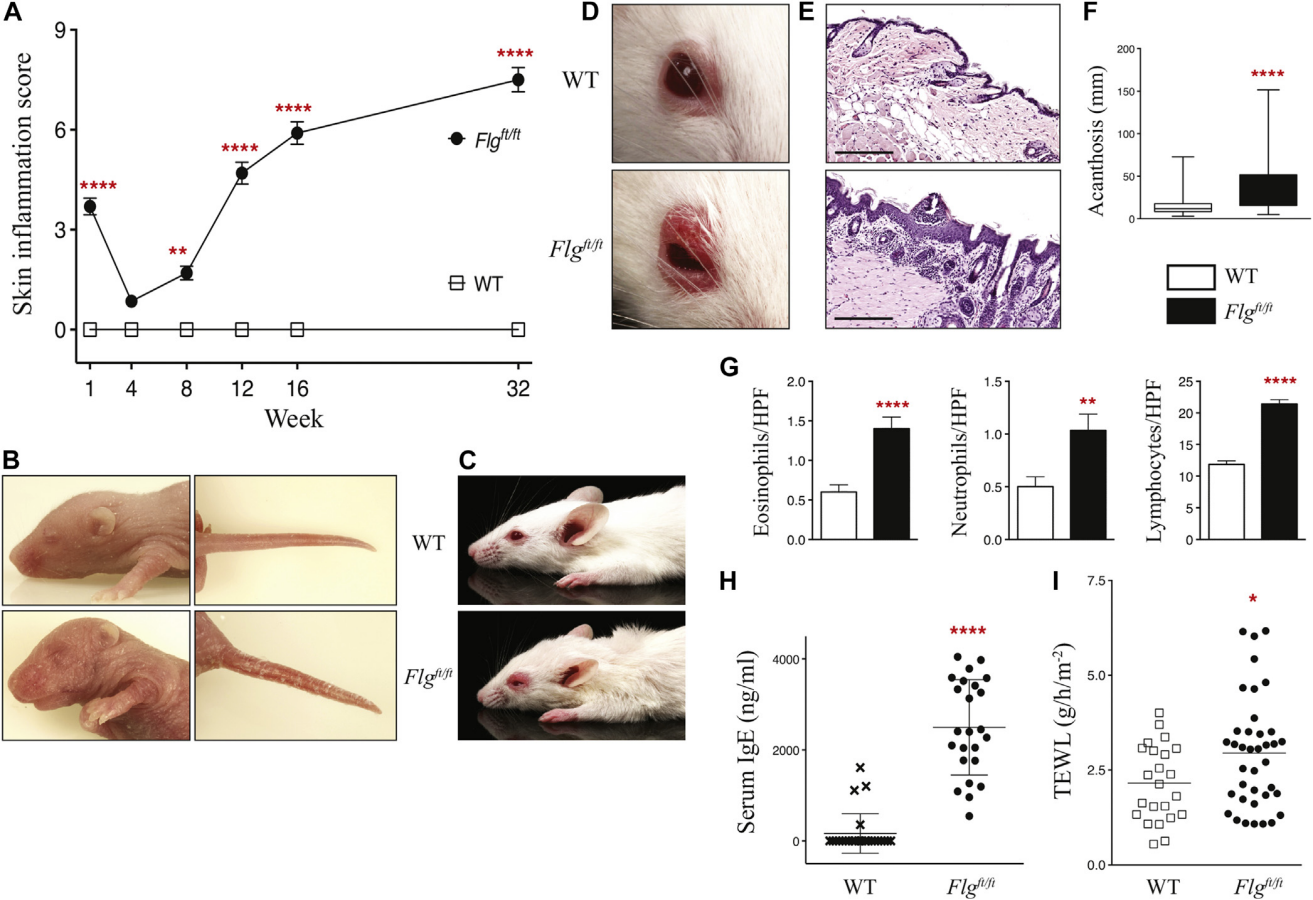
Development of dermatitis and atopy in filaggrin-deficient mice. **A,** Macroscopic clinical scoring of *Flg*^*ft/ft*^ versus WT mice. Data are from 25 to 30 mice per strain (scored longitudinally). Statistical significance was determined with 2-way ANOVA. **B,** Gross phenotype of a representative *Flg*^*ft/ft*^ neonate in comparison with a WT littermate. **C,** Gross phenotype of *Flg*^*ft/ft*^ and WT mice (age matched at 12 weeks). **D,** Representative image of the eczematous inflammation that develops in the eyelid skin of *Flg*^*ft/ft*^ mice at 12 weeks. **E,** Representative hematoxylin and eosin–stained biopsy specimens of eyelid skin from 12-week-old *Flg*^*ft/ft*^ and WT mice. *Scale bar* = 200 μm. **F** and **G**, Epidermal acanthosis scoring (Fig 1, *F*) and dermal eosinophil, neutrophil, and lymphocyte counts per high-power field (*HPF*; Fig 1, *G*) in lesional skin. Data are from 6 to 10 mice per strain. **H,** Total serum IgE levels from adult mutant and 12-week-old age-matched WT mice. **I,** Transepidermal water loss *(TEWL)* at 12 weeks. Data are from 23 to 38 mice per strain. *P < .05, **P < .01, and ****P < .0001.

Single mutant *Flg*^*ft*^ congenic mice without the *Matt*^*ma*^ mutation were generated (see Fig E1 in this article's Online Repository at www.jacionline.org) on the proallergic BALB/c background.^17^, ^18^, ^19^ *Flg*^*ft/ft*^ mice have attenuated profilaggrin expression in the epidermis and absent functional filaggrin monomer (see Fig E2 in this article's Online Repository at www.jacionline.org), which is similar to what is seen in *FLG*-null patients.^5^ As neonates, *Flg*^*ft/ft*^ mice spontaneously have marked ichthyosis-like dermatitis with edema, erythema, hyperlinearity, and scaling compared with WT control animals (Fig 1, *A* and *B*). Longitudinal clinical scoring of skin inflammation shows that the early ichthyosis-like dermatitis observed in neonatal *Flg*^*ft/ft*^ mice dissipates by 4 weeks, with significant (*P* < .01) spontaneous eczematous-like dermatitis developing in *Flg*^*ft/ft*^ mice from 8 weeks (Fig 1, *A* and *C*). By 12 weeks, all *Flg*^*ft/ft*^ mice have overt dermatitis, with eczematous lesions occurring initially in eyelid skin (Fig 1, *A* and *D*). The dermatitis, which is characterized by edema, erythema, scaling, and lichenification (Fig 1, *D*), progresses with age to excoriation and severe pathology, with pruritic erythematous lesions progressing beyond the eyelid skin to around the eye and rostrum at 32 weeks (see Fig E3, *A*, in this article's Online Repository at www.jacionline.org). Histopathologic analysis of skin at 12 weeks demonstrates profound acanthosis (*P* < .0001) in *Flg*^*ft/ft*^ mice (Fig 1, *F*), and significant infiltration of eosinophils (*P* < .0001), neutrophils (*P* < .01), and lymphocytes (*P* < .0001) into the dermis (Fig 1, *G*). By 32 weeks, an increasing incidence of erythema and edema is evident in tail skin (see Fig E3, *B*) and the ear pinnae (see Fig E3, *C*), indicating a spectrum of pathology at these sites. Ear histopathology in *Flg*^*ft/ft*^ mice (see Fig E3, *C*) shows significantly increased acanthosis (see Fig E3, *D*) and inflammatory cell infiltrates in the dermis (data not shown). Thus *Flg*^*ft/ft*^ mice on a BALB/c background spontaneously have ichthyosis as neonates and frank eczematous dermatitis in adulthood.

### Filaggrin-deficient mice are atopic with an altered immunologic cutaneous environment

An increased IgE level is a cardinal marker of AD.^20^ *Flg*^*ft/ft*^ mice had significantly (*P* < .0001) increased serum IgE levels at 12 weeks (Fig 1, *H*), indicating AD-like dermatitis. Addressing skin barrier integrity, the significantly (*P* < .05) increased transepidermal water loss^21^ demonstrated skin barrier dysregulation in *Flg*^*ft/ft*^ mice (Fig 1, *I*). By using NF-κB reporter mice, NF-κB activation was observed in the skin of *Flg*^*ft/ft*^*NF-κB–Luc* neonates (see Fig E3, *E*), and the level of NF-κB activation was significantly increased in nonlesional skin of 12-week-old adult *Flg*^*ft/ft*^*NF-κB–Luc* mice (see Fig E3, *F* and *G*). Furthermore, *Flg*^*ft/ft*^ mice have significantly increased contact hypersensitivity skin inflammation (*P* < .01) in response to oxazolone hapten^22^ at a dose evoking limited skin inflammation in WT mice (see Fig E4 in this article's Online Repository at www.jacionline.org). Therefore filaggrin deficiency leads to a defective skin barrier, with subclinical cutaneous inflammation in nonlesional skin, and is accompanied by a lower threshold for skin inflammation after exposure to hapten.

**Fig 2:**
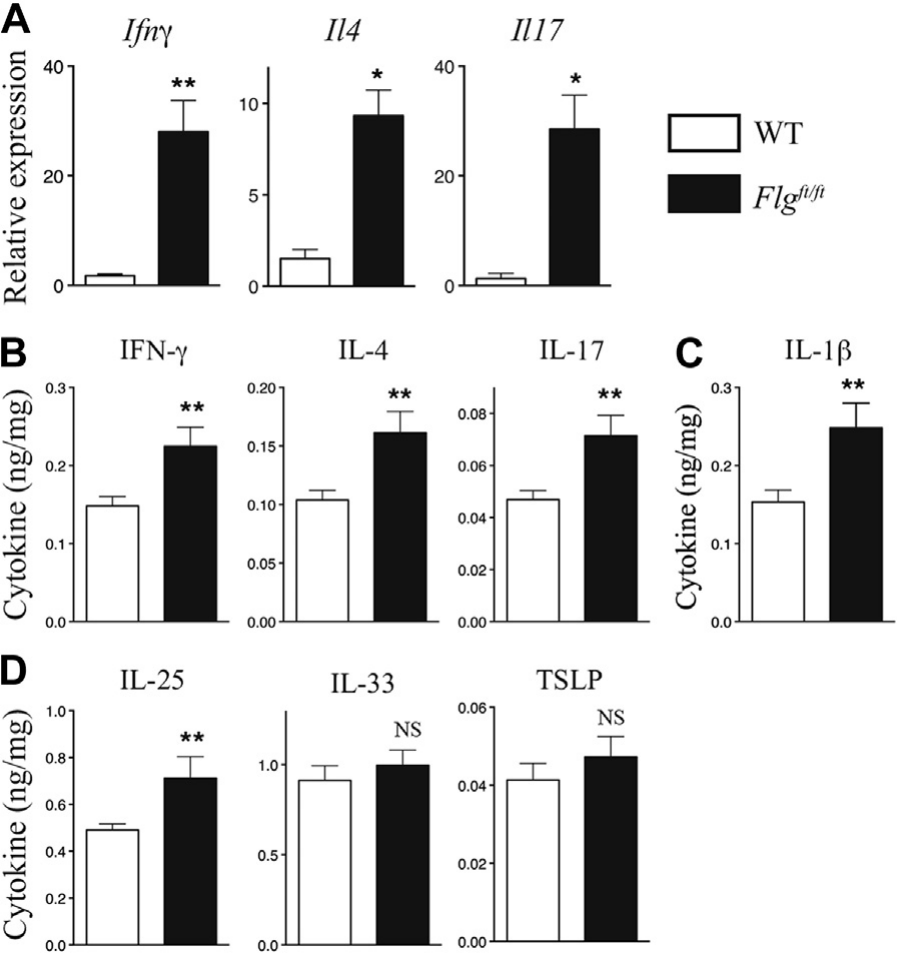
Skin inflammation in filaggrin-deficient mice. **A,** Fold change in *Ifng*, *Il4*, and *Il17* mRNA expression (n = 6-8 per group) in skin. Data are representative of 3 experiments. **B-D,** Cytokine quantification in nonlesional skin expressed as nanograms of cytokine per milligram of protein. Data are from 25 to 35 mice per strain. Statistical significance was determined with the Student *t* test. *P < .05 and **P < .01. *NS*, Nonsignificant.

Gene expression analysis of lesional eyelid skin in 12-week-old *Flg*^*ft/ft*^ mice (Fig 2, *A*) demonstrated increased *Ifng*, *Il4*, and *Il17* transcripts, which are typical of mixed type 1, 2, and 17 cutaneous cytokine responses in lesional inflamed skin. Given the dysregulated skin barrier and increased NF-κB activity in the uninvolved skin of *Flg*^*ft/ft*^ mice, the basal inflammatory state of nonlesional skin was addressed by quantifying cytokines in *Flg*^*ft/ft*^ and WT skin (Fig 2, *C-E*, and see Fig E5 in this article's Online Repository at www.jacionline.org). In nonlesional *Flg*^*ft/ft*^ skin there was a significant (*P* < .01) approximately 50% upregulation in the levels of IL-4, IL-17, and IFN-γ (Fig 2, *B*) in addition to IL-1β (*P* < .01; Fig 2, *C*). Because the alarmin cytokines IL-25, IL-33, and thymic stromal lymphopoietin (TSLP) are implicated in the pathogenesis of allergic skin inflammation in experimental models and patients with AD,^16^, ^23^, ^24^, ^25^, ^26^ we evaluated alarmin expression in *Flg*^*ft/ft*^ mice. IL-25 was significantly (*P* < .05) upregulated in *Flg*^*ft/ft*^ skin, whereas IL-33 and TSLP levels were not (Fig 2, *D*). There were no differences between *Flg*^*ft/ft*^ and WT mice for other cytokines assayed (see Fig E5). These data demonstrate that filaggrin-deficient mice spontaneously become atopic, with dysregulated skin barrier function. *Flg*^*ft/ft*^ mice have cutaneous subclinical inflammation characterized by a generalized immune response with increased cardinal T_H_1, T_H_2, and T_H_17 cytokine levels, as well as selective upregulation of IL-1β and IL-25 in nonlesional skin.

### Filaggrin-deficient mice have an expansion of type 2 innate lymphoid cells in the skin

**Fig 3:**
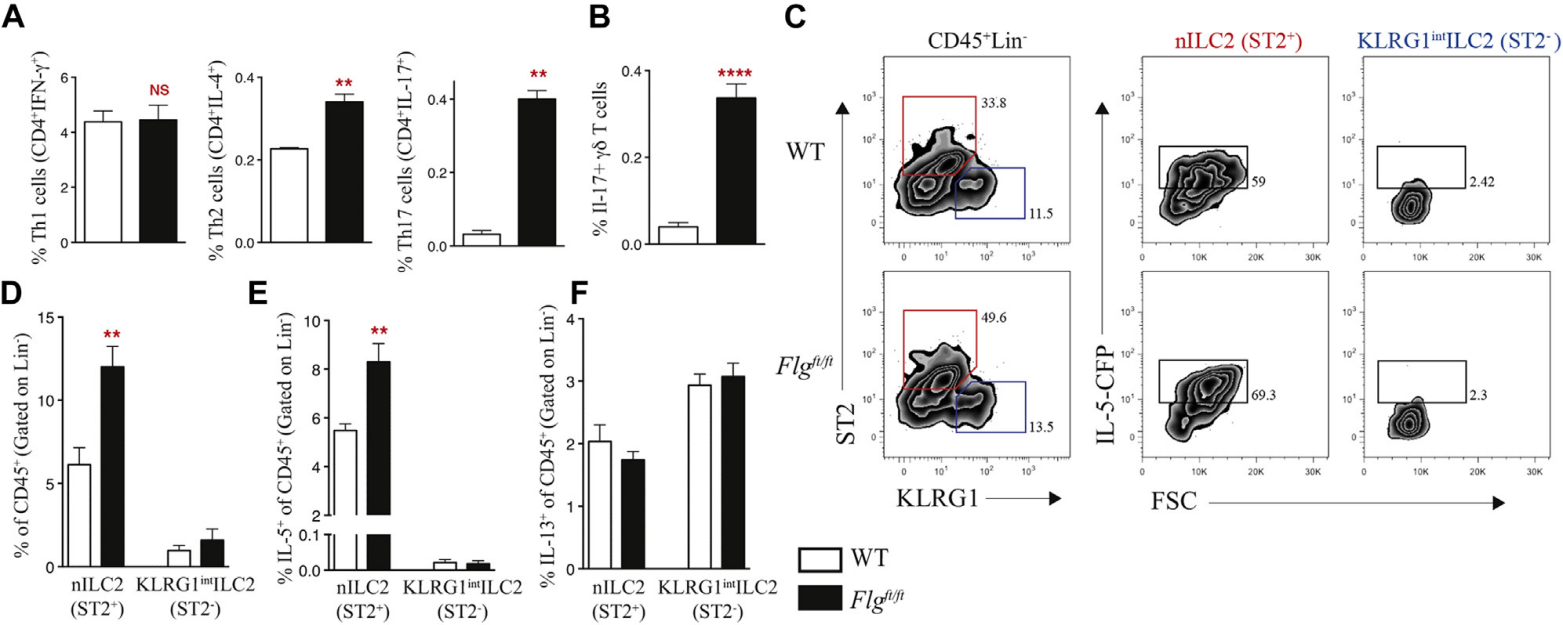
Filaggrin deficiency leads to an increase in ILC2 numbers in the skin. **A,** Frequency of T_H_1 (CD4^+^IFN-γ^+^), T_H_2 (CD4^+^IL-4^+^), and T_H_17 (CD4^+^IL-17^+^) cells as a percentage of total cells in skin dLNs. **B,** Frequency of IL-17^+^ γδ T cells as a percentage of total cells in skin dLNs. **C,** Expression of ST2 and KLRG1 on CD45^+^Lin^−^ cells in the skin; *outlined areas* indicate nILC2s *(red)* or KLRG1^int^ ILC2s *(blue)*. **D,** Frequency of nILC2s (ST2^+^) and KLRG1^int^ ILC2s (ST2^−^) of CD45^+^ cells (gated on Lin^−^ cells) between *Flg*^*ft/ft*^ and WT mice. **E,** Frequency of IL-5^+^ nILC2s (ST2^+^) and IL-5^+^ KLRG1^int^ ILC2s (ST2^−^) of CD45^+^ cells (gated on Lin^−^ cells) between *Flg*^*ft/ft*^ and WT mice. **F,** Frequency of IL-13^+^ nILC2s (ST2^+^) and IL-13^+^ KLRG1^int^ ILC2 (ST2^−^) of CD45^+^ cells (gated on Lin^−^ cells) between *Flg*^*ft/ft*^ and WT mice. Data are representative of 3 experiments (n = 6-10 mice per group). Statistical significance was determined with the Student *t* test. **P < .01 and ****P < .0001. *NS*, Nonsignificant.

Because IFN-γ, IL-4, and IL-17 levels were increased in the skin of *Flg*^*ft/ft*^ mice, we examined the cellular source of these cytokines. IFN-γ^+^CD4^+^ T_H_1 cell frequency in the draining lymph nodes (dLNs) was comparable between *Flg*^*ft/ft*^ and WT mice (Fig 3, *A*). Generating dual *Flg*^*ft/ft*^-IL-4–KN2^11^ reporter mice demonstrated a significantly (*P* < .01) increased frequency of T_H_2 cells in *Flg*^*ft/ft*^ mice (Fig 3, *A*). The use of *Flg*^*ft/ft*^IL-17–enhanced green fluorescent protein (eGFP) reporter mice showed increased frequency (*P* < .01) of both IL-17–eGFP^+^CD4^+^ T_H_17 cells (Fig 3, *A*) and IL-17–eGFP^+^ γδ T cells (Fig 3, *B*) in the dLNs of *Flg*^*ft/ft*^ mice.

The alterations in type 2 and type 17 responses led us to examine the role of the recently described innate lymphoid cells, which were classified as negative for lineage markers and expressing IL-7Rα (CD127), CD25, and CD90, which have been investigated in a number of inflammatory diseases.^27^, ^28^, ^29^ ILC2s are implicated in allergy; are regulated by IL-25, IL-33, and TSLP; are characterized by expression of the transcription factors GATA3 and retinoic acid–related orphan receptor (ROR) α; and produce the type 2 cytokines IL-5, IL-9, and IL-13.^27^, ^29^, ^30^ Recently ILC2 numbers have been shown to be increased in the skin of patients with AD and also in mouse skin after chemical (MC903)– and allergen (house dust mite)–elicited cutaneous inflammation.^16^, ^25^ In the skin of WT mice, numbers of resident ILC2s (Lin^−^ST2^+^KLRG1^lo^IL-7Rα^+^Thy-1^lo^Sca-1^hi^; see Fig E6, *A*, in this article's Online Repository at www.jacionline.org) correspond to the natural type 2 innate lymphoid cell (nILC2) classification that has recently been defined as distinct from the IL-25–elicited inflammatory type 2 innate lymphoid cell (iILC2) population in the lung.^31^ In the skin of *Flg*^*ft/ft*^ mice, there is a significant (*P* < .01) increase in the frequency of nILC2s compared with the frequency in WT animals (Fig 3, *C* and *D*). In addition, there is a KLRG1^int^ ILC2 in mouse skin (see Fig E6, *A*), which lacks ST2 expression consistent with iILC2s^31^; however, iILC2s are KLRG1^hi^ and are a distinct population. There was no difference in the frequency of KLRG1^int^ ILC2s in *Flg*^*ft/ft*^ mice compared with WT mice (Fig 3, *C* and *D*). Consistent with the absence of the iILC2s in the skin of *Flg*^*ft/ft*^ mice, we also do not see this population after 4 days of treatment of WT ear skin with MC903 (see Fig E7, *A*, in this article's Online Repository at www.jacionline.org). However, as reported,^31^ after 3 days of intraperitoneal treatment with recombinant IL-25 but not IL-33, iILC2 numbers are increased in the lungs of WT mice (see Fig E7, *B*). In contrast to the lung, after 3 days of intradermal treatment in ear skin with recombinant IL-25, there is a negligible influx of iILC2s (see Fig E7, *C*), indicating that iILC2s might not be upregulated in the skin on inflammation.

Previously, a study demonstrated, by using quantitative RT-PCR, that activated skin ST2^+^ ILC2s produced more IL-5, leading to eosinophil influx and development of spontaneous dermatitis.^26^ We have generated a novel IL-5–cerulean fluorescent protein (CFP) reporter mouse to accurately quantify IL-5–expressing ILC2s in the skin (see Fig E8, *A-C*, in this article's Online Repository at www.jacionline.org). In WT mice nILC2s in the skin produce IL-5 in the steady state (see Fig E6, *B* and *C*). Strikingly, having generated *Flg*^*ft/ft*^IL-5–CFP reporter mice, there was a significant (*P* < .01) increase in the frequency of IL-5–producing nILC2s in *Flg*^*ft/ft*^ mice compared with levels in WT control animals (Fig 3, *C* and *E*). In addition, numbers of nILC2s (see Fig E9, *A*, in this article's Online Repository at www.jacionline.org) and IL-5–producing nILC2s (see Fig E9, *B*) were also increased in the skin dLNs of *Flg*^*ft/ft*^ relative to levels in WT mice. Because ILC2s express IL-13,^12^ IL-13–eGFP reporter mice were used to look at IL-13–expressing ILC2s in the skin. In the skin of WT mice, both nILC2s and KLRG1^int^ ILC2s constitutively express IL-13 in the steady state, with KLRG1^int^ ILC2s having marginally higher IL-13 expression (see Fig E6, *D*). However, *Flg*^*ft/ft*^IL-13–eGFP reporter mice demonstrated no differences in the frequency of IL-13–producing nILC2s and KLRG1^int^ ILC2s between *Flg*^*ft/ft*^ and WT mice (Fig 3, *F*). Using *Flg*^*ft/ft*^IL-17–eGFP reporter mice, we examined whether the marked increase in IL-17–producing CD45^+^ cells in the skin of *Flg*^*ft/ft*^ mice (see Fig E9, *C*) correlated with an increase in IL-17 production by innate lymphoid cells. We found no IL-17–producing population in *Flg*^*ft/ft*^ mice (see Fig E9, *D*). No differences were observed in numbers of ILC3s (Lin^−^IL-7Rα^+^ST2^−^RORγT^+^) in the skin (see Fig E9, *E*). Similarly, there were no differences in numbers of ILC1s (Lin^−^IL-7Rα^+^ T-box transcription factor [T-bet]^+^; see Fig E9, *F*). Using *Flg*^*ft/ft*^KN2 IL-4 reporter mice, we investigated whether increased numbers of IL-4–producing CD45^+^ cells in the skin of *Flg*^*ft/ft*^ mice (see Fig E9, *G*) corresponded to increased numbers of IL-4–producing ILC2s. No ILC2 population produced IL-4 in the skin of *Flg*^*ft/ft*^ mice (see Fig E9, *H*). In addition to IL-5–producing nILC2 expansion, there was significantly increased skin infiltration of eosinophils (*P* < .05; non-B/non-T-cells [NBNT] cells SiglecF^+^CD11b^+^), mast cells (*P* < .01; NBNT ckit^+^FcεR1^+^ cells), and basophils (*P* < .0001; NBNT FcεR1^+^ckit^−^ cells) in *Flg*^*ft/ft*^ mice (see Fig E9, *I*). Collectively, *Flg*^*ft/ft*^ mice on a BALB/c background have a cutaneous expansion of IL-5–producing nILC2s, with a mixed type 2 and type 17 inflammatory milieu.

### Filaggrin-deficient mice have spontaneous pulmonary inflammation

**Fig 4:**
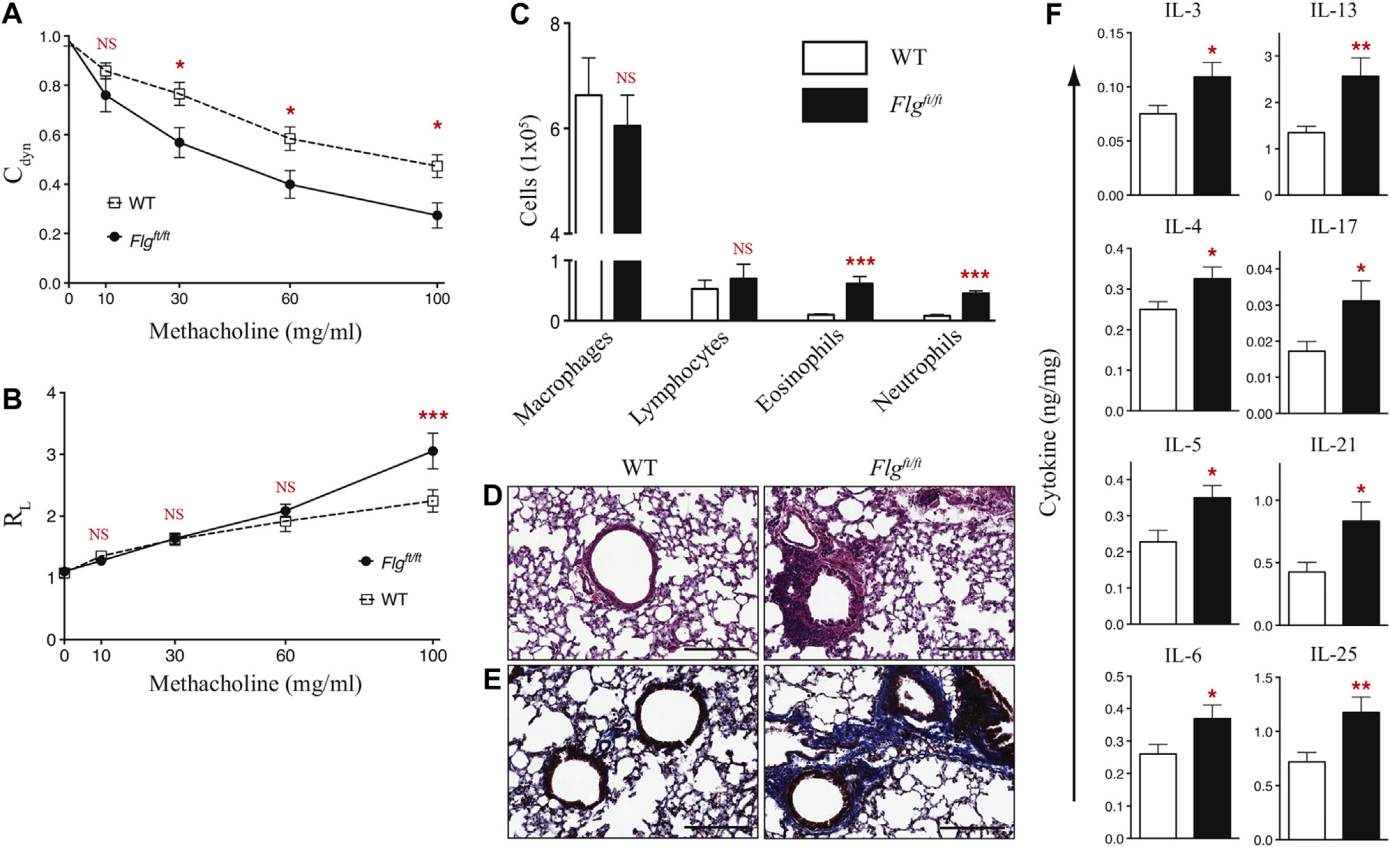
Spontaneous lung inflammation in filaggrin-deficient mice. **A** and **B**, Measurement of AHR as assessed by C_dyn_ (Fig 4, *A*) and R_L_ (Fig 4, *B*) in response to increasing doses of methacholine. Data are representative of 3 experiments (n = 6-8 mice per group). Statistical significance was determined with 2-way ANOVA. **C,** Differential cell counts from bronchoalveolar lavage fluid. Data are representative of 3 experiments (n = 6-8 mice per group). Statistical significance was determined with the Student *t* test. **D** and **E**, Representative hematoxylin and eosin–stained (Fig 4, *D*) and Masson trichrome–stained (Fig 4, *E*) lung tissue. *Scale bars* = 200 μm. **F,** Cytokine quantification in the lung expressed as nanograms of cytokine per milligram of protein. Data are from 16 to 20 mice per strain. Statistical significance was determined with the Student *t* test. *P < .05, **P < .01, and ***P < .001. *NS*, Nonsignificant.

*FLG* mutations in human subjects predispose to asthma development after AD occurrence, exemplifying the atopic march.^7^, ^8^ Therefore we analyzed AHR in *Flg*^*ft/ft*^ mice. Similar to studies on flaky tail mice,^9^ 16-week-old *Flg*^*ft/ft*^ mice had AHR comparable with that seen in WT control animals, with no pulmonary inflammation (data not shown) despite having dermatitis (Fig 1, *A*). When analyzing 32-week-old *Flg*^*ft/ft*^ mice with marked dermatitis, significantly altered C_dyn_ was observed (Fig 4, *A*). *Flg*^*ft/ft*^ mice had no differences in R_L_ apart from at the highest methacholine concentration (Fig 4, *B*). Significant changes in dynamic lung compliance, but not resistance in *Flg*^*ft/ft*^ mice, suggests that aberrant lung function is predominately caused by peripheral alterations, such as lung parenchyma elasticity, with lesser effects on central airway function.^32^ In agreement with this altered lung function, there were significantly (*P* < .01) more cells in bronchoalveolar lavage fluid of *Flg*^*ft/ft*^ mice (see Fig E10, *A*, in this article's Online Repository at www.jacionline.org), with a significant (*P* < .001) increase in neutrophil and eosinophil numbers (Fig 4, *C*).

The compromised lung function in *Flg*^*ft/ft*^ mice older than 24 weeks was reflected in significant lung pathology with mixed peribronchial cellular infiltrates observed in hematoxylin and eosin–stained sections (Fig 4, *D*). *Flg*^*ft/ft*^ mice did not have goblet cell hyperplasia, peribronchial eosinophilia, or marked airway occlusion (data not shown). Consistent with altered peripheral changes to the lungs of *Flg*^*ft/ft*^ mice, there was marked collagen deposition (Fig 4, *E*), with significantly increased (*P* < .05) collagen levels in the lungs of *Flg*^*ft/ft*^ mice (see Fig E10, *B*). Quantification of pulmonary eosinophil peroxidase and myeloperoxidase enzymatic activity (see Fig E10, *C* and *D*) indicated increased eosinophil and neutrophil activity in the lungs of *Flg*^*ft/ft*^ mice, which is in agreement with the increased numbers of eosinophils and neutrophils in bronchoalveolar lavage fluid (Fig 4, *C*). With respect to increased eosinophil numbers in the skin and lungs of deficient mice, we also noted inflammation in the upper esophagus of *Flg*^*ft/ft*^ mice (see Fig E11, *A*, in this article's Online Repository at www.jacionline.org) with significant (*P* < .05) eosinophil infiltration (see Fig E11, *B*). However, *Flg*^*ft/ft*^ mice do not have the overt esophageal pathology reported in food allergen–induced models of eosinophilic esophagitis.^33^

In the inflamed lungs of *Flg*^*ft/ft*^ mice, levels of the type 2 cytokines IL-4, IL-5, and IL-13 were significantly (*P* < .05-.01) increased (Fig 4, *F*). IL-17 levels were significantly (*P* < .05) increased in lung homogenates (Fig 4, *F*), as were levels of IL-3 (*P* < .05), IL-6 (*P* < .05), and IL-21 (*P* < .05). We also observed an increase in IL-25 levels in the lungs of *Flg*^*ft/ft*^ mice (Fig 4, *F*), whereas levels of the other epithelial cytokines (ie, IL-33 and TSLP) were unchanged (see Fig E10, *E*). Levels of other cytokines assayed were comparable in the lungs of WT and *Flg*^*ft/ft*^ mice (see Fig E10, *E*). These data demonstrate the development of marked pulmonary inflammation with age in *Flg*^*ft/ft*^ mice on a BALB/c background secondary to dermatitis development, with decreased lung compliance, increased parenchymal collagen deposition, and eosinophil and neutrophil infiltration with mixed type 2 and type 17 pulmonary inflammation.

### Cutaneous inflammation occurs in filaggrin-deficient mice in the absence of adaptive immunity

**Fig 5:**
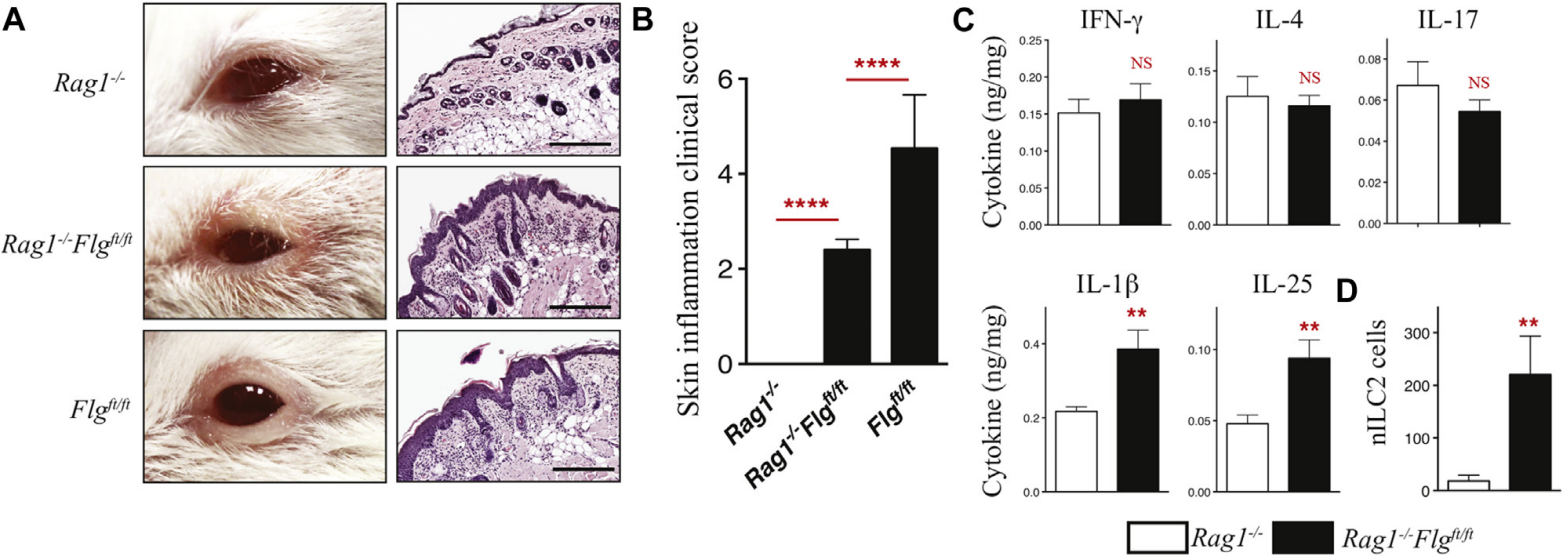
Dermatitis develops in filaggrin-deficient mice independent of adaptive immunity. **A,** Representative images of inflammation in eyelid skin and representative hematoxylin and eosin–stained eyelid skin. *Scale bars* = 200 μm. **B,** Clinical scoring of eyelids at 12 weeks. Data are from 20 to 25 mice per strain. Statistical significance was determined with the Student *t* test. **C,** Cytokine quantification in nonlesional skin expressed as nanograms of cytokine per milligram of protein. Data are from 16 to 20 mice per strain. Statistical significance was determined with the Student *t* test. **D,** nILC2 numbers in the skin. Data are representative of 3 experiments (n = 6-10 mice per group). Statistical significance was determined with the Student *t* test. **P < .01 and ****P < .0001. *NS*, Nonsignificant.

To assess the relative role of innate versus adaptive immunity in the context of spontaneous skin and lung inflammation caused by filaggrin deficiency, we crossed *Flg*^*ft/ft*^ and *Rag1*^*−/*−^ mice, generating T cell– and B cell–deficient *Rag1*^*−/*−^*Flg*^*ft/ft*^ mice. Neonatal *Rag1*^*−/*−^*Flg*^*ft/ft*^ mice retain the erythematous scaly skin phenotype typical of *Flg*^*ft/ft*^ mice. Adult *Rag1*^*−/*−^*Flg*^*ft/ft*^ mice had eczematous eyelid lesions similar to *Flg*^*ft/ft*^ mice (Fig 5, *A*), with a significant clinical score (Fig 5, *B*). Histopathology reveals that *Rag1*^*−/*−^*Flg*^*ft/ft*^ mice have marked inflammation (Fig 5, *A*), with significantly increased epidermal acanthosis, and infiltration of eosinophils (*P* < .001) and neutrophils (*P* < .01) into the dermis (see Fig E12, *A*, in this article's Online Repository at www.jacionline.org) relative to *Rag1*^*−/*−^ mice.

Cytokine protein levels were assessed in nonlesional skin biopsy specimens of *Rag1*^*−/*−^*Flg*^*ft/ft*^ mice. Consistent with the cutaneous innate immune milieu observed in the skin of *Flg*^*ft/ft*^ mice, both IL-1β and IL-25 levels were significantly (*P* < .01) upregulated in nonlesional skin of *Rag1*^*−/*−^*Flg*^*ft/ft*^ mice relative to those in *Rag1*^*−/*−^ control skin (Fig 5, *C*). However, IL-4, IL-17, and IFN-γ levels were unchanged in the skin (Fig 5, *C*), as were IL-33 and TSLP levels (see Fig E12, *B*). No increases were observed in the levels of other cytokines assayed (see Fig E12, *B*). Importantly, inflammation observed in *Rag1*^*−/*−^*Flg*^*ft/ft*^ mice is associated with a significant (*P* < .01) cutaneous nILC2 expansion relative to that seen in *Rag1*^*−/*−^ mice (Fig 5, *D*). *Rag1*^*−/*−^*Flg*^*ft/ft*^ mice do not have lung inflammation, as measured based on compliance and resistance (see Fig E13, *A* and *B*, in this article's Online Repository at www.jacionline.org) and other parameters (data not shown), as observed in *Flg*^*ft/ft*^ mice. Although pulmonary IL-1β levels were increased (*P* < .05) in *Rag1*^*−/*−^*Flg*^*ft/ft*^ mice, there were no differences in the levels of other cytokines analyzed (see Fig E13, *C*).

Adult *Rag1*^*−/*−^*Flg*^*ft/ft*^ mice had eczematous eyelid lesions with significant clinical scoring (Fig 5, *B*) but no lung inflammation (see Fig E13, *A-C*). *Rag1*^*−/*−^*Flg*^*ft/ft*^ mice were reconstituted with B and T cells to address whether adaptive immunity exacerbated skin or lung inflammation. B cell– and T cell–reconstituted *Rag1*^*−/*−^*Flg*^*ft/ft*^ mice have more severe dermatitis relative to that seen in *Rag1*^*−/*−^*Flg*^*ft/ft*^ mice (see Fig E14, *A*, in this article's Online Repository at www.jacionline.org), with significantly increased clinical scores (see Fig E14, *B*). Furthermore, reconstituted *Rag1*^*−/*−^*Flg*^*ft/ft*^ mice had marked atopy, which was significantly greater than that seen in *Rag1*^*−/*−^ mice receiving B and T cells (see Fig E14, *C*). In addition to more marked skin inflammation, B cell– and T cell–reconstituted *Rag1*^*−/*−^*Flg*^*ft/ft*^ mice had compromised lung function with a specific significant alteration in C_dyn_, indicating progression to secondary lung inflammation (see Fig E14, *D*). B cell– and T cell–reconstituted *Rag1*^*−/*−^*Flg*^*ft/ft*^ mice had no differences in R_L_ (see Fig E14, *E*). These data demonstrate that the spontaneous development of dermatitis caused by filaggrin deficiency is mediated by innate immunity involving upregulation of IL-1β, IL-25, and nILC2s, with adaptive immunity required for the development of severe skin pathology and progression to lung inflammation.

### ILC2s are expanded in the skin of patients with *FLG* mutations

**Fig 6:**
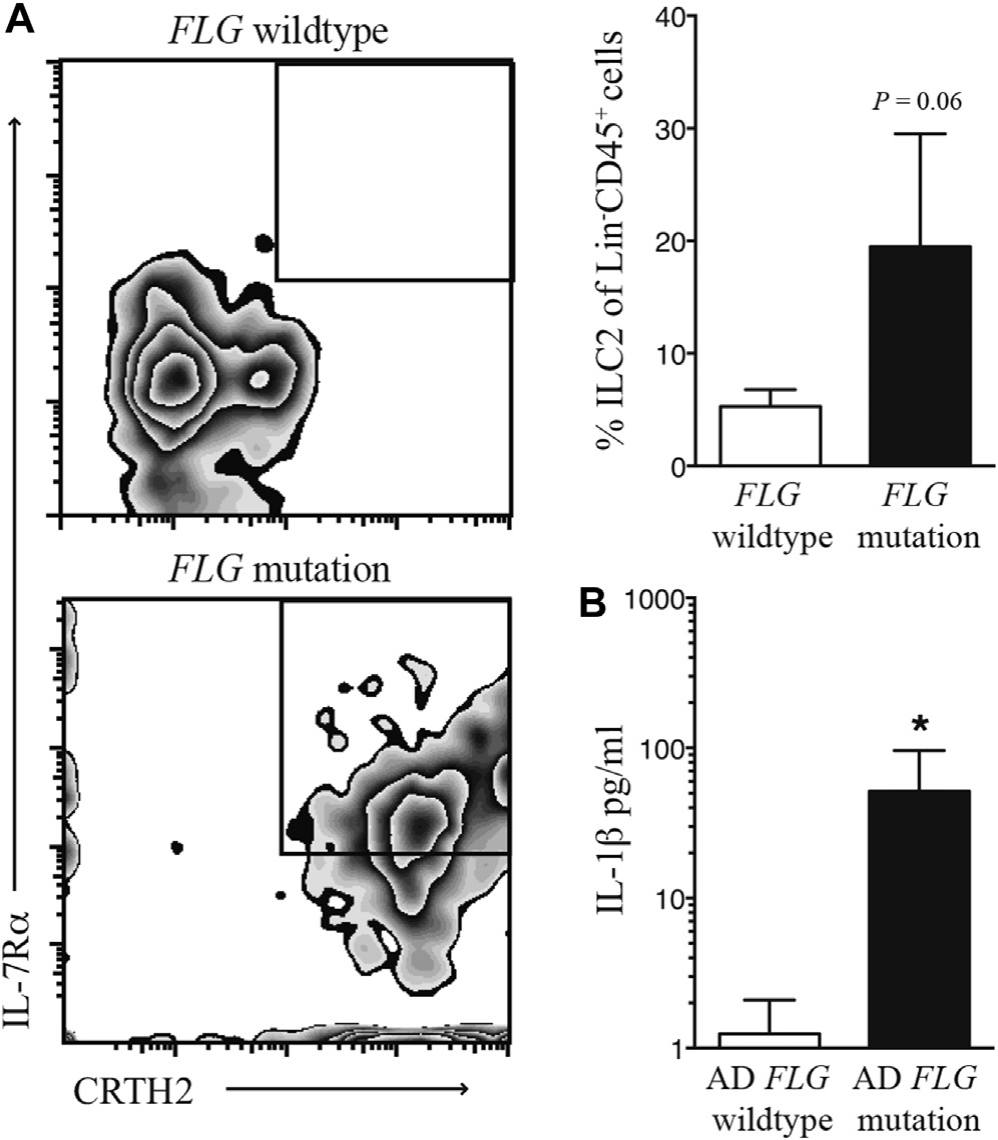
ILC2s are expanded in the skin of patients with *FLG* mutations. **A,** Chemoattractant receptor–homologous molecule expressed on T_H_2 lymphocytes *(CRTH2)*^+^IL-7Rα^+^ ILC2s (gated on Lin^−^CD45^+^ cells) are upregulated in skin suction blisters of patients with *FLG* mutations in comparison with those of patients without *FLG* mutations. **B,** IL-1β is upregulated in the blister fluid of acute lesional skin from patients with AD with *FLG* mutations compared with those without *FLG* mutations. Statistical significance was determined with the Mann-Whitney test. *P < .05.

It has been reported recently that ILC2s are present in the skin of patients with AD.^16^, ^25^ Given the spontaneous expansion in ILC2 frequency in the skin of filaggrin-deficient mice (Fig 3), we investigated whether ILC2 frequency was altered in the skin of patients with mutations in *FLG*. We now show that there are increased ILC2 numbers (*P* = .06) in skin blisters taken from nonlesional skin of patients with *FLG* mutations^5^ compared with the skin of *FLG* WT subjects (Fig 6, *A*). Furthermore, similar to the increase in IL-1β levels in the skin of filaggrin-deficient mice (Fig 2), IL-1β levels are significantly upregulated within the blister fluid of acute lesional skin from patients with moderate-to-severe AD with *FLG* mutations compared with levels seen in those without *FLG* mutations (Fig 6, *B*).

## Discussion

Filaggrin mutations have been identified as the major genetic predisposer to AD development and in the context of the atopic march, the subsequent progression to AD-associated asthma. We now show that filaggrin-deficient mice, which have a mutation analogous to the filaggrin mutations found in human subjects, are atopic, have spontaneous AD-like inflammation, and progress to pulmonary inflammation with age. Emerging evidence from genome-wide association studies and Immunochip and transcriptome analyses^34^, ^35^, ^36^, ^37^, ^38^, ^39^ has highlighted the complexity of genetic predisposition in human AD. The polygenetic nature of dermatitis is also evident in mice, with marked differences between BALB/c and C57BL/6J strains in the magnitude of skin inflammation and the functional genes involved.^16^, ^25^ Herein filaggrin-deficient mice were generated on a BALB/c background, a strain predisposed to type 2/type 17–associated inflammation.^17^, ^18^, ^19^, ^40^, ^41^, ^42^ BALB/c *Flg*^*ft/ft*^ mice have spontaneous AD-like skin inflammation and pulmonary inflammation unlike the filaggrin mutant on the C57BL/6J background^10^ and the *Flg* deletion knockout C57BL/6 mouse.^43^ These contrasting phenotypes in mice, highlighting the gene-modifying effects of strain background, are indicative of the complexity of how loss-of-function *FLG* mutations in human subjects can lead to AD.

*Flg*^*ft/ft*^ mice have neonatal ichthyosis, with eczematous AD inflammation developing with 100% penetrance in *Flg*^*ft/ft*^ mice by 12 weeks. Skin barrier dysregulation in the nonlesional skin of *Flg*^*ft/ft*^ mice, as evidenced by transepidermal water loss, reflects the epidermal barrier dysfunction that has been shown in nonlesional skin in patients with AD.^44^, ^45^ Furthermore, the increased susceptibility of *Flg*^*ft/ft*^ mice to contact hypersensitivity inflammation indicates that the dysregulated barrier can facilitate allergenic sensitization. Eczematous inflammation in filaggrin-deficient mice is characterized by increased type 1, type 2, and type 17 cytokines, indicating a generalized inflammatory response in lesional skin. Expansion of T_H_2 and T_H_17 cells in skin dLNs and IL-17–eGFP^+^ γδ T cells indicates that the mixed inflammatory cutaneous milieu resembles the dual T_H_2 and T_H_17 T-cell response in patients with AD.^46^ Importantly, development of spontaneous lesions in *Rag1*^*−/*−^*Flg*^*ft/ft*^ mice demonstrates that dermatitis can occur in the absence of adaptive immunity. However, we demonstrate that adaptive immunity is required for progression to secondary lung inflammation. A recent study demonstrated that *Rag2*^*−/*−^*Flg*^*ft/ft*^ mice (homozygous for *Matt*^*ma*^) did not have dermatitis in the dorsal flank.^47^ Differences in terms of the development of skin inflammation between both studies may be due to the presence of the matted mutation *(Matt*^*ma*^*)*, which is still present in the *Rag2*^*−/*−^*/Flg*^*ft/ft*^ mice in the study by Leisten et al,^47^ confounding direct comparison with our findings, where only the *Flg*^*ft*^ mutation is present in mice; animal housing conditions might also be a factor. However, similar to our findings, this letter reports an increase in the frequency of Lin^−^CD3^−^Thy1^+^IL7R^+^ innate lymphoid cells in *Rag2*^*−/−*^*Flg*^*ft/ft*^ mice in comparison with that in *Rag2*^*−/*−^ control animals.^47^

Increased NF-κB activity in nonlesional skin of *Flg*^*ft/ft*^ mice indicates basal subclinical cutaneous inflammation. Indeed, although relatively modest, the increased IL-4, IL-17, and IFN-γ protein levels in nonlesional skin demonstrated a generalized subclinical inflammatory milieu in the skin. Nonlesional skin was assessed to investigate inflammatory mechanisms in the barrier-dysregulated skin of *Flg*^*ft/ft*^ mice, avoiding potential complications of secondary inflammation associated with lesional skin. These data correlate with recent transcriptomic studies analyzing the uninvolved skin of patients with AD and filaggrin mutations, which demonstrated upregulation of T_H_1- and T_H_2-associated transcripts.^39^ Importantly, upregulation of IL-1β in the nonlesional skin of both *Flg*^*ft/ft*^ and *Rag1*^*−/*−^*Flg*^*ft/ft*^ mice is consistent with our previous work in which epidermal IL-1β levels were increased in *Flg*^*ft/ft*^ mice and also in patients with AD with filaggrin mutations,^48^ indicating a key role for IL-1β in the dysregulated cutaneous environment arising from filaggrin deficiency. Interestingly, we now show that IL-1β levels are upregulated in the blister fluid of acute lesional skin of patients with AD with *FLG* mutations in comparison with those in patients with AD with WT *FLG*.

IL-25 and IL-33 are overexpressed in skin of patients with AD,^16^, ^49^, ^50^, ^51^ with TSLP overexpression associated with skin inflammation in mice.^52^ *Flg*^*ft/ft*^ and *Rag1*^*−/*−^*Flg*^*ft/ft*^ mice have an increase in IL-25 levels, but not IL-33 and TSLP levels, in nonlesional skin. Recent studies have shown roles for IL-25, IL-33, and TSLP in eliciting ILC2s during cutaneous inflammation in transgenic mice after IL-2 treatment in response to chemical and allergen challenge and in the skin of patients with AD.^16^, ^24^, ^25^, ^26^ Previously, it has been demonstrated that dermal ILC2s in the steady state constitutively produce IL-13, but on activation, this population expanded and switched to a proinflammatory phenotype characterized by increased *Il5* mRNA expression, which promoted eosinophil infiltration and spontaneous dermatitis.^26^ Importantly, we now show a specific upregulation of these activated IL-5–producing ILC2s in the skin of *Flg*^*ft/ft*^ mice using a novel IL-5–CFP reporter mouse. These ILC2s, which also constitutively express IL-13, correspond to the nILC2s described by Huang et al.^31^ After intraperitoneal recombinant IL-25 treatment, we also observe an increase in the lungs of the KLRG1^hi^ iILC2 population recently described,^31^ but we do not observe this population in the skin of *Flg*^*ft/ft*^ mice or in ear skin of WT mice after IL-25 or MC903 treatment.

Further work is needed to define the expansion of distinct ILC2 subpopulations in different organs. Strikingly, the ILC2 expansion in filaggrin-deficient mice translates to patients, with ILC2 frequency increased in skin suction blisters of patients with *FLG* mutations compared with that seen in those without *FLG* mutations. Collectively, the increased frequency of ILC2s in the skin of human subjects with *FLG* mutations is comparable with the phenotype that develops spontaneously in filaggrin-deficient mice. Importantly, nILC2 numbers are also specifically increased in the skin of *Rag1*^*−/*−^*Flg*^*ft/ft*^ mice, indicating the importance of ILC2s in the pathogenesis of skin inflammation arising from skin barrier dysregulation caused by filaggrin deficiency. Indeed, *Rag1*^*−/*−^ mice have dermatitis associated with ILC2 activity after treatment with IL-2–JES6-1.^26^ In addition to ILC2 expansion, we also observed an increase in the numbers of eosinophils, mast cells, and basophils in the skin of *Flg*^*ft/ft*^ mice, which is similar to the phenotype seen with IL-2–JES6-1–induced inflammation.^26^

Carriers of filaggrin mutations have an increased risk of AD-associated asthma.^7^, ^8^ Filaggrin-deficient mice have a striking age-dependent progression to pulmonary inflammation characterized by compromised lung function and involving parenchymal alterations in lung physiologic dynamics. Decreased compliance in filaggrin-deficient mice was associated with increased collagen deposition and eosinophil and neutrophil infiltration of the lungs with mixed type 2 and type 17 inflammatory responses, reflecting aspects of pulmonary pathology associated with multiple asthma phenotypes.^53^

Importantly, *Rag1*^*−/*−^*Flg*^*ft/ft*^ mice do not have lung pathology, demonstrating that the adaptive immune response is required for the progression from dermatitis to pulmonary inflammation.

In summary, filaggrin deficiency in mice leads to the development of features of the atopic march that occur in patients with AD with *FLG* mutations. This study highlights how skin inflammation in the context of dysregulated skin barrier function develops independently of the adaptive immune response, whereas the subsequent progression to compromised lung function requires adaptive immunity.

**Key messages**

• Filaggrin-deficient mice have spontaneous AD-like inflammation and progress to compromised pulmonary function, reflecting the atopic march in patients with AD.
• AD-like inflammation in the context of filaggrin deficiency is associated with a cutaneous expansion in IL-5–producing ILC2 numbers in mice, and in patients with AD with *FLG* mutations, there is an increase in ILC2 infiltration of the skin.
• In the absence of adaptive immunity, filaggrin-deficient mice experience spontaneous skin inflammation but do not have lung pathology.

## Supplementary data

Online Repository Data

Table E1

Fig E1

Fig E2

Fig E3

Fig E4

Fig E5

Fig E6

Fig E7

Fig E8

Fig E9

Fig E10

Fig E11

Fig E12

Fig E13

Fig E14
