# Supplementary material for: Spontaneous atopic dermatitis is mediated by innate immunity, with the secondary lung inflammation of the atopic march requiring adaptive immunity
Source: J Allergy Clin Immunol. 2016 Feb;137(2):482–91. doi: 10.1016/j.jaci.2015.06.045 (PMC4735016; doi:10.1016/j.jaci.2015.06.045)
Supplement: Online Repository Data [file mmc1.docx]

**ONLINE REPOSITORY: Revised Submission JACI-D-14-01528**

**Spontaneous atopic dermatitis is mediated by innate immunity with the secondary lung inflammation of the atopic march requiring adaptive immunity.**

**METHODS**

**Protein isolation for filaggrin western blotting.** Dorsal skin epidermis was separated from the dermis after incubation in PBS containing 5mM EDTA at 50°C for 5 minutes, followed by rapid cooling in ice-cold PBS ([1](#_ENREF_1), [2](#_ENREF_2)). Epidermal protein isolation was performed in 0.5mL in urea/Tris buffer containing protease inhibitors (‘Halt’ Pierce Thermo, UK), by several strokes with an IKA T10 Basic Ultra-Turrax homogenizer ([2](#_ENREF_2)). Homogenates were centrifuged at 13000rpm for 15 minutes at 4^o^C. Filaggrin was detected using a Rabbit Polyclonal Antibody (Covance, Co Nr. PRB-417P in 1/1000 dilution) and Polyclonal Goat Anti-Rabbit Immunoglobulin HRP-conjugated secondary antibody (Dako, Co Nr. P0448 in 1/3000 dilution).

**Immunohistochemistry.** Immunohistochemistry was performed on skin and esophagus sections at 12- and 32-weeks, respectively. Sections were blocked with 20% normal goat serum, incubated overnight at 4°C. Skin and esophagus sections were incubated with Filaggrin anti-rabbit antibody (Covance), and rat anti-mouse MBP (gifted by Prof J. Lee, Mayo Clinic, Scottsdale, AZ) ([3](#_ENREF_3)) respectively, before being quenched with 0.3% hydrogen peroxidase. Skin sections were incubated with HRP conjugated goat anti-rabbit immunoglobulin (Dako), and esophagus sections with biotinylated anti-rat IgG (ABC kit, Vector Laboratories). Sections were stained with diaminobenzidin (DAB kit, Dako) and counterstained with hematoxylin. On esophageal sections, eosinophils were enumerated per high-power field (HPF), and the average taken from 20 HPFs.

**Histology.** Tissue sections were fixed in 10% formal saline and embedded in paraffin wax. Dorsal skin sections, ear skin and eyelid sections, from 12-week and 32-week time-points in the longitudinal study, were stained with hematoxylin and eosin. Lung sections from 32-week mice were stained with hematoxylin and eosin, and Masson’s trichrome for collagen quantification. All histology sections were examined in a blinded fashion by two observers independently. To quantify the individual inflammatory cells infiltrating the skin, we used a previous described scoring system ([4](#_ENREF_4)). We counted the total number of cells per 1,000 high-power fields (HPF) of view, with 20 HPFs scored per mouse ([2](#_ENREF_2)). Acanthosis was quantified by measuring epidermal thickness across 20 HPFs per mouse.

**Antibody ELISA.** Total serum IgE was measured in serum from mice at the12-week time-point in the longitudinal study, using sandwich ELISA according to the manufacturer's instructions (BD Pharmingen). Bound IgE was detected using monoclonal biotin-labeled detection antibodies and streptavidin HRP (BD Pharmingen).

**RNA isolation and real-time PCR.** RNA was isolated from skin at the 12-week time-point in the longitudinal study by using RNeasy kit (Qiagen, UK) and reverse transcribed with the Quantitect reverse transcription kit incorporating a genomic DNA elimination step (Qiagen). Real-time quantitative PCR was performed on an AB StepOnePLus Real-time PCR system (Life Technologies, UK), using predesigned TaqMan gene expression assays specific for murine *Ifnγ* (Mm01168134_m1), *Il4* (Mm00445259_m1) and *Il17a* (Mm00439618_m1). Specific gene expression was normalized to murine glyceraldehyde-3-phosphate dehydrogenase (VIC probe, 4352339E; Life Technologies, UK). Fold expression was calculated by using the comparative cycle threshold method of analysis and is presented as relative quantification. Data expressed as relative quantification were calculated and compared with glyceraldehyde- 3-phosphate dehydrogenase as a housekeeping gene.

**Protein extraction from skin and lung for cytokine ELISA measurement.** For ELISA measurement of bioavailable cytokines in the non-lesional skin, proteins were isolated as described ([5](#_ENREF_5)). Briefly, skin tissue was excised from mice at the 12-week time-point in the longitudinal study and homogenized with 0.5 mL extraction buffer (containing 10 mM Tris pH 7.4, 150 mM NaCl, 1% Triton X-100, and 5 mM EDTA) containing protease inhibitors (‘Halt’ Pierce Thermo, UK), using an IKA T10 Basic Ultra-Turrax homogenizer. The homogenates were centrifuged at 13000 rpm for 15 min. Lungs were homogenized in a buffer containing 1 X PBS, 2% foetal bovine serum and 0.5% cetyltrimethylammonium bromide. Cytokines in samples were determined using ELISA kits per the manufacturer’s instructions (R&D Systems, Minneapolis, MN). All cytokine levels in individual samples were normalized to the total protein following BCA assay.

**Trans-epidermal water loss (TEWL).** A Courage and Khazaka Tewameter TM210 (Enviroderm, Evesham, UK) was used for measurement of TEWL on 12-week mice ([2](#_ENREF_2), [4](#_ENREF_4)). TEWL assessment was performed on the dorsal skin site at least 24 hours after hair clipping, and 24 hours prior to euthanasia and the analyses of other parameters. Hair clipping did not induce any visual damage to the skin. TEWL was recorded at ambient temperature 19-21^°^C and humidity 50% ± 5.

**Bioluminescence.** *In situ* bioluminescent imaging was performed in neonates and 12-week adult *NF-κB-Luc* transgenic luciferase reporter mice as previously described with some modifications ([6](#_ENREF_6)), with light emission from the skin detected as photon counts over a defined area expressed as a Regions of Interest (ROI) ([7](#_ENREF_7)). Since luciferase production is under the control of the NF-κB promoter, luciferase activity represents NF-*κ*B activation, and the value of the value of ROI represents the density of luciferase activity ([7](#_ENREF_7)). Briefly, imaging of transgenic mice was performed with an IVIS Lumina Series III Pre-Clinical In Vivo imaging system (Perkin Elmer, UK). D-Luciferin (150 mg/kg; Perkin Elmer, UK) dissolved in PBS, was injected i.p. to preanesthetized mice. Immediately afterward, the mice were placed in a light-sealed chamber connected to an ultra-sensitive camera consisting of an image intensifier coupled to a CCD camera. The mice were maintained under anesthesia provided by isofluorane inhaled through nose cones in the chamber during imaging. The pseudo colored images represent light intensity.

**Contact hypersensitivity models.** Contact hypersensitivity was performed as described ([8](#_ENREF_8)), with some modifications. The 0.02% challenge dose of Ox was applied once to the inner and outer surfaces of the left ear after initial Ox sensitization (2% Ox) on the dorsal flank, of 8-10 week mice. Vehicle was applied to the right ear of sensitized mice as a control. Ear thickness was measured 2 hours after treatment. Ear swelling is presented relative to vehicle treated ears.

**Generating IL-5-cerulean fluorescent protein(CFP) reporter mice.** To generate the IL-5 reporter mouse, the Cerulean fluorescent protein gene was inserted directly after the start codon of the Il5 gene (Fig E8). A 9 kb region of the murine Il5 gene centred on exon 1 was amplified from mouse BAC RPCI23-449-D15 using PCR primers ASEQ 4751 and ASEQ 4752 (Table E1), then digested with *BamHI* and *SpeI*. The digested PCR product was then ligated into the pl2XR plasmid digested with *BamHI* and *XbaI* to create pl2XR + IL5. pl2XR was prepared in dam- dcm- *E. coli* for digestion with *XbaI*. The Cerulean gene cassette was amplified from the pl452 Cerulean plasmid with PCR primers ASEQ 4818 and ASEQ 4819 for recombineering into pl2XR + IL5. ASEQ 4818 consisted of the 50 bp upstream of the start codon of the Il5 gene as a homologous region, followed by a 28 bp sequence homologous to the 5’ end of the Cerulean gene cassette. ASEQ 4819 consisted of the first 50 bp of the Il5 gene coding sequence as a homologous region, followed by a *HindIII* site for Southern blot screening and a 28 bp sequence homologous to the 3’ end of the Cerulean gene cassette. Recombineering was used to insert the Cerulean gene cassette into the *Il5* 9kb region. The pl2XR + IL5 plasmid and the Cerulean gene cassette PCR product were sequentially transfected into SW106 *E. coli* cells and kanamycin resistant SW106 colonies were cultured and sequenced across the entire insert. The pl2XR + IL5 + Cerulean plasmid was transfected into CCB ES cells and antibiotic resistant ES colonies were picked and expanded. The ES cell DNA was screened for targeted integration of the IL-5Cerulean gene cassette by Southern blot analysis. From 750 clones screened, 3 positive clones were identified and 1 positive clone (AG7) was expanded and injected into C57BL/6 blastocysts to generate chimaeric mice. The chimeras were further crossed and genotyped for germline transmission of the IL-5Cerulean gene. Genotyping was performed on ear biopsies of pups using the PCR primers ASEQ 5358 and ASEQ 5376 (Table E1), yielding a wildtype product of 351 bp and a targeted product of 3.0 kb. IL-5Cerulean mice were crossed with BALB/c deletor mice to remove the floxed neomycin resistance gene cassette.

**Cell preparation and flow cytometric analysis of skin draining lymph nodes and ear tissue.** Skin draining lymph nodes were collected from mice at the 12-week time-point in the longitudinal study, and cells isolated for flow-cytometry analyses. Ear tissue was excised and incubated overnight at 4 degrees in 3mg/mL Dispase (Sigma, UK) in Hank’s Buffered Salt Solution (Sigma, UK). Ear tissue was then removed from dispase and incubated for 1 hour at 37 degrees in an orbital incubator in 1mg/mL Collagenase D (Roche, UK). Lung tissue was minced and incubated for 1 hour at 37 degrees in an orbital incubator in 1mg/mL Collagenase D (Roche, UK). Single-cell suspensions were prepared from all the organs and erythrocytes lysed with Lysis solution (BD, UK) where needed. For surface staining, single-cell suspensions were prepared in flow-cytometry buffer (PBS, 2% FCS, 0.05% sodium azide, and 0.5 mmol/L EDTA). LIVE/DEAD Fixable Aqua Dead Cell Stain Kit (Life Technologies, UK) was used to determine cell viability. Cells were blocked using Fc-block CD16/CD32 (2.4G2;BD Biosciences). Directly conjugated antibodies with FITC, PE, PE-Texas Red, PerCP, PerCP-Cy5.5, PeCy7, V450, APC and APC-Cy7 were used. Murine cells were stained with BD Biosciences mAbs; CD2 (RM2-5), CD4 (RM4-5), CD8 (53-6.7), CD11b (M170), CD11c (HL3), CD19 (ID3), CD25 (7D4), CD44 (IM7), CD45.2 (104), CD90.2 (53-2.1), CD103 (M290), CD132 (4G3), c-kit (2B8), FceR1 (MAR-1), F4/80 (BM8), Gr-1 (RB6-85C), ICOS (7E.17G9), Integrin-β7 (FIB504), IL-7Rα (A7R34), KLRG1 (2F1), Siglec-F (E50-2440), Sca-1 (D7), TCR-β (H57-597), eBiosciences mAbs; CD3 (17A2), CD69 (H1.2F3) NK1.1 (PK136), NKG2D (CX5), BioLegend mAb; TCR γδ (GL3), MD biosciences mAb; T1/ST2-FITC (DJ8), and R&D mAb; IL-17BR (FAB1040F). For intracellular cytokine, staining cells were cultured with 50 ng/mL phorbol 12-myristate 13-acetate and 500 ng/mL ionomycin in the presence of 10 μg/mL Brefeldin A for 4 h. Permeabilized cells were stained with eBiosciences mAb; IFN-γ (XMG1.2). For intranuclear transcription factor expression, cells were stained with RORγT (Q31-378) and Tbet (O4-46). Cells were acquired using a Cyan (ADP Analyzer; Beckman Coulter). Gating of cells was based on the specific isotype control values as well as fluorochrome minus 1 setting when needed. For intracellular staining, cells were cultured for 5 hours at 1 x 10^6^ cells/mL in RPMI 1640 (Invitrogen) supplemented with 10% heat-inactivated FCS (Labtech), 2 mmol/L L-glutamine (Invitrogen), and 50 U/mL penicillin and 50 mg/mL streptomycin (Invitrogen), stimulated with phorbol 12-myristate 13-acetate (PMA)/ionomycin 25ng/mL and 250 ng/mL, respectively. Brefeldin A (10 mg/mL; Sigma-Aldrich) was added to cells one hour prior to the end of culture. Surface markers were stained first, followed by intracellular/intranuclear antibodies or matched isotype controls. IL-4 expression in the cells was identified in IL-4-KN2 reporter mice ([9](#_ENREF_9)), using Biolegend mAb; huCD2-biotin (TS1/8). All analyses were performed using FlowJo software (Tree Star Inc).

**Treatment with IL-25 or IL-33.** For the assessment of ILC2s in the lung, 8-10 week wild-type mice were treated daily for three consecutive days with intraperitoneal injection with PBS or 500 ng of IL-25 (R&D Systems) or IL-33 (Biolegend, San Diego, USA). Mice were sacrificed on day 4 and single cell suspensions of lung cells was prepared as previously described ([10](#_ENREF_10)). For the assessment of ILC2s in the ear, mice were treated by intradermal injection in one ear daily for three consecutive days with PBS or 500 ng of IL-25 or IL-33 as indicated. Mice were sacrificed 24 hours after the last treatment, and ear tissue excise tissue skin derived single cell suspension was prepared as described.

**MC903 Topical Application.** MC903 (calcipotriol; Tocris) was dissolved in EtOH and topically applied on the ears of 8-10 week wild-type mice (4 nmol in 25μl per ear), daily for 4 consecutive days ([11](#_ENREF_11)). As vehicle control, the same volume of EtOH was applied on mouse ears. Mice were sacrificed 24 hours after the last treatment, and ear tissue excise tissue skin derived single cell suspension was prepared as described.

**Bronchoalveolar lavage.** Bronchoalveolar lavage (BAL) fluid was collected following AHR analyses in 32-week mice, by cannulating the trachea, and lavaging the lungs twice with 0.8mL ice-cold PBS ([12](#_ENREF_12)). BAL cells were pelleted, washed, and counted. For BAL cell cytospins, the numbers of eosinophils, neutrophils, macrophages, and lymphocytes was determined by performing a differential count, with at least 400 cells per slide, on Giemsa-stained cytocentrifuge preparations.

**Collagen, EPO and MPO assays.** Briefly, lung tissue from the 32-week mice following AHR and BAL was collected and homogenized, followed by over-night acid-pepsin collagen extraction at 4^0^C. Total soluble collagen content of the mixture was then determined with a Sircol Collagen Assay kit (Biocolor, Carrickfergus, UK) ([10](#_ENREF_10)), as described by the manufacturer. Sample collagen concentrations were interpolated from absorbance measurement at 555 nm against a standard curve, and expressed against protein in the lung, μg/mg, following BCA quantification. For EPO and MPO ELISA, lungs were homogenized in a buffer containing 1 X PBS, 2% foetal bovine serum and 0.5% cetyltrimethylammonium bromide. EPO and MPO activity was detected using *O*-phenylenediamine in 50mM HEPES (with 6mM KBr and 2mM Resorcinol), and TMB as substrates respectively. Absorbance was measured at 490nm for EPO and 450nm for MPO, with activity interpolated from a standard curves (Enzo Life Sciences, UK), and expressed against protein in the lung, U/mg.

**Immune cell reconstitution of *Rag1^-/-^* and *Rag1^-/-^Flg^ft/ft^* mice.** *Rag1^-/-^* and *Rag1^-/-^Flg^ft/ft^* mice were reconstituted with T and B lymphocytes from spleens of wild-type mice. Spleens were removed and passed through 70-μm-pore-size sieves to prepare single-cell suspensions. Splenocytes were depleted of erythrocytes by lysis with PharmLyseR (BD Biosciences). For B cell enrichment, cells were labeled with B220 microbeads (Miltenyi Biotec) before magnetic separation via AutoMACSTM Pro according manufacturer’s instructions (Miltenyi Biotec). For T cell enrichment, APC-conjugated antibody against CD3 mAb was used, before incubating with the anti-APC microbeads for magnetic separation via AutoMACSTM Pro. Purity was determined by flow cytometry at > 90% for B cells and at > 90 % for T cells. Recipient *Rag1^-/-^* or *Rag1^-/-^Flg^ft/ft^* mice were then injected intravenously with 1 x 10^7^ B cells and 6 x 10^6^ T cells in 100μl PBS.

**Genotyping for *FLG* mutations.** The mutations R501X and 2282del4 ([13](#_ENREF_13)) were genotyped using Taqman allelic discrimination assays (Life Technologies), as previously described ([14](#_ENREF_14)). R501X was screened using forward primer 5’ CAC TGG AGG AAG ACA AGG ATC G 3’, reverse primer 5’ CCC TCT TGG GAC GCT GAA 3’ and the probes VIC-CAC GAG ACA GCT C and 6-FAM-CAT GAG ACA GCT CC. 2282del4 was screened using forward primer 5’ CCA CTG ACA GTG AGG GAC ATT CA 3’, reverse primer 5’ GGT GGC TCT GCT GAT GGT GA 3’ and the probes 6-FAM- CAC AGT CAG TGT CAG GCC ATG GAC A and VIC-AGA CAC ACA GTG TCA GGC CAT GGA CA alleles. Assays were performed in 384-well plates with each reaction comprising of 20ng DNA, 2.5 µl Universal PCR master mix and 0.125 µl 40X assay mix in a final reaction volume of 6 µl. Assays were run on an Applied Biosystems 7900HT Fast Real-Time PCR system under the following conditions: 1 cycle at 50°C for 2 minutes followed by 1 cycle at 95°C for 10 minutes then 40 cycles of 95°C 15 sec; 60°C 1 minute. Samples heterozygous for the 2282del4 mutation were also confirmed by Sanger sequencing using the published primers RPT1P7 (5' – AAT AGG TCT GGA CAC TCA GGT - 3') and RPT2P1 (5' – GGG AGG ACT CAG ACT GTT T - 3') ([15](#_ENREF_15)). PCR conditions were 94°C for 5 min; 35 cycles of 94°C for 40 s, 57°C for 1 min, 72°C for 2 min; final extension step at 72°C for 7 min. PCR clean up and sequencing was performed by Source Bioscience plc.

**REFERENCES FOR ONLINE METHODS**

1. Presland RB, Boggess D, Lewis SP, Hull C, Fleckman P, Sundberg JP. Loss of normal profilaggrin and filaggrin in flaky tail (ft/ft) mice: an animal model for the filaggrin-deficient skin disease ichthyosis vulgaris. The Journal of investigative dermatology. 2000;115(6):1072-81.

2. Fallon PG, Sasaki T, Sandilands A, Campbell LE, Saunders SP, Mangan NE, et al. A homozygous frameshift mutation in the mouse Flg gene facilitates enhanced percutaneous allergen priming. Nat Genet. 2009;41(5):602-8.

3. Furuta GT, Nieuwenhuis EE, Karhausen J, Gleich G, Blumberg RS, Lee JJ, et al. Eosinophils alter colonic epithelial barrier function: role for major basic protein. American journal of physiology Gastrointestinal and liver physiology. 2005;289(5):G890-7.

4. Saunders SP, Goh CS, Brown SJ, Palmer CN, Porter RM, Cole C, et al. Tmem79/Matt is the matted mouse gene and is a predisposing gene for atopic dermatitis in human subjects. J Allergy Clin Immunol. 2013;132(5):1121-9.

5. Guinea-Viniegra J, Zenz R, Scheuch H, Hnisz D, Holcmann M, Bakiri L, et al. TNFalpha shedding and epidermal inflammation are controlled by Jun proteins. Genes & development. 2009;23(22):2663-74.

6. Carlsen H, Moskaug JO, Fromm SH, Blomhoff R. In vivo imaging of NF-kappa B activity. J Immunol. 2002;168(3):1441-6.

7. Hams E, Saunders SP, Cummins EP, O'Connor A, Tambuwala MT, Gallagher WM, et al. The hydroxylase inhibitor dimethyloxallyl glycine attenuates endotoxic shock via alternative activation of macrophages and IL-10 production by B1 cells. Shock. 2011;36(3):295-302.

8. Smith P, Fallon RE, Mangan NE, Walsh CM, Saraiva M, Sayers JR, et al. Schistosoma mansoni secretes a chemokine binding protein with antiinflammatory activity. The Journal of experimental medicine. 2005;202(10):1319-25.

9. Mohrs K, Wakil AE, Killeen N, Locksley RM, Mohrs M. A two-step process for cytokine production revealed by IL-4 dual-reporter mice. Immunity. 2005;23(4):419-29.

10. Amu S, Saunders SP, Kronenberg M, Mangan NE, Atzberger A, Fallon PG. Regulatory B cells prevent and reverse allergic airway inflammation via FoxP3-positive T regulatory cells in a murine model. J Allergy Clin Immunol. 2010;125(5):1114-24 e8.

11. Zhang Z, Hener P, Frossard N, Kato S, Metzger D, Li M, et al. Thymic stromal lymphopoietin overproduced by keratinocytes in mouse skin aggravates experimental asthma. Proceedings of the National Academy of Sciences of the United States of America. 2009;106(5):1536-41.

12. Mangan NE, Dasvarma A, McKenzie AN, Fallon PG. T1/ST2 expression on Th2 cells negatively regulates allergic pulmonary inflammation. European journal of immunology. 2007;37(5):1302-12.

13. Palmer CN, Irvine AD, Terron-Kwiatkowski A, Zhao Y, Liao H, Lee SP, et al. Common loss-of-function variants of the epidermal barrier protein filaggrin are a major predisposing factor for atopic dermatitis. Nat Genet. 2006;38(4):441-6.

14. Kezic S, O'Regan GM, Yau N, Sandilands A, Chen H, Campbell LE, et al. Levels of filaggrin degradation products are influenced by both filaggrin genotype and atopic dermatitis severity. Allergy. 2011;66(7):934-40.

15. Smith FJ, Irvine AD, Terron-Kwiatkowski A, Sandilands A, Campbell LE, Zhao Y, et al. Loss-of-function mutations in the gene encoding filaggrin cause ichthyosis vulgaris. Nat Genet. 2006;38(3):337-42.
