# Supplementary material for: Spontaneous atopic dermatitis is mediated by innate immunity, with the secondary lung inflammation of the atopic march requiring adaptive immunity
Source: J Allergy Clin Immunol. 2016 Feb;137(2):482–91. doi: 10.1016/j.jaci.2015.06.045 (PMC4735016; doi:10.1016/j.jaci.2015.06.045)
Supplement: Table E1 [file mmc2.docx]

**Table E1: Primers used in the generation of IL-5-CFP mice**

| Name | Sequence | Purpose |
| --- | --- | --- |
| ASEQ 4751 | GCTAGTTAATGGATCCGCCAGGAATATAGGCGTTAGGCACC | BamHI + IL-5 HA Forward |
| ASEQ 4752 | CACTTCATGAACTAGTGGCAATGGTGCATGTCTGTAACCTC | SpeI + IL-5 HA Reverse |
| ASEQ 4818 | tgctctgcgctcttcctttgctgaaggccagcgctgaagacttcagagtcgccaccatggtgagcaagggcgaggagc | IL-5 HA + Cerulean Recombineering Forward |
| ASEQ 4819 | AgctgaaggaataagttatactttacctcattgcttgtcaacagagctcgAAGCTTtagaactagtggatcccctcgagggacc | IL-5 HA + HindIII + Cerulean Recombineering Reverse |
| ASEQ 4905 | cagggacccagaggagagactgagt | 3’ IL-5Cerulean probe Forward |
| ASEQ 4906 | ggatctggctatttctgtctccca | 3’ IL-5Cerulean probe Reverse |
| ASEQ 5358 | CAGAGAGAGAATAAATTGCTTGGGG | IL-5Cerulean Genotype Forward |
| ASEQ 5376 | CATAGGTAAGGAGATCTGGTAGGG | IL-5Cerulean Genotype Reverse |
| ASEQ 863 | CTTGGGTGGAGAGGCTATTC | Neomycin Forward |
| ASEQ 864 | AGGTGAGATGACAGGAGATC | Neomycin Reverse |
| ASEQ 2270 | TACCTGGCCTGGTCTGGACACAGTG | Cre Forward |
| ASEQ 2271 | ATGGCTAATCGCCATCTTCCAGCAG | Cre Reverse |
