# Supplementary figures and images for: Spontaneous atopic dermatitis is mediated by innate immunity, with the secondary lung inflammation of the atopic march requiring adaptive immunity
Source: J Allergy Clin Immunol. 2016 Feb;137(2):482–91. doi: 10.1016/j.jaci.2015.06.045 (PMC4735016; doi:10.1016/j.jaci.2015.06.045)

Figure E1

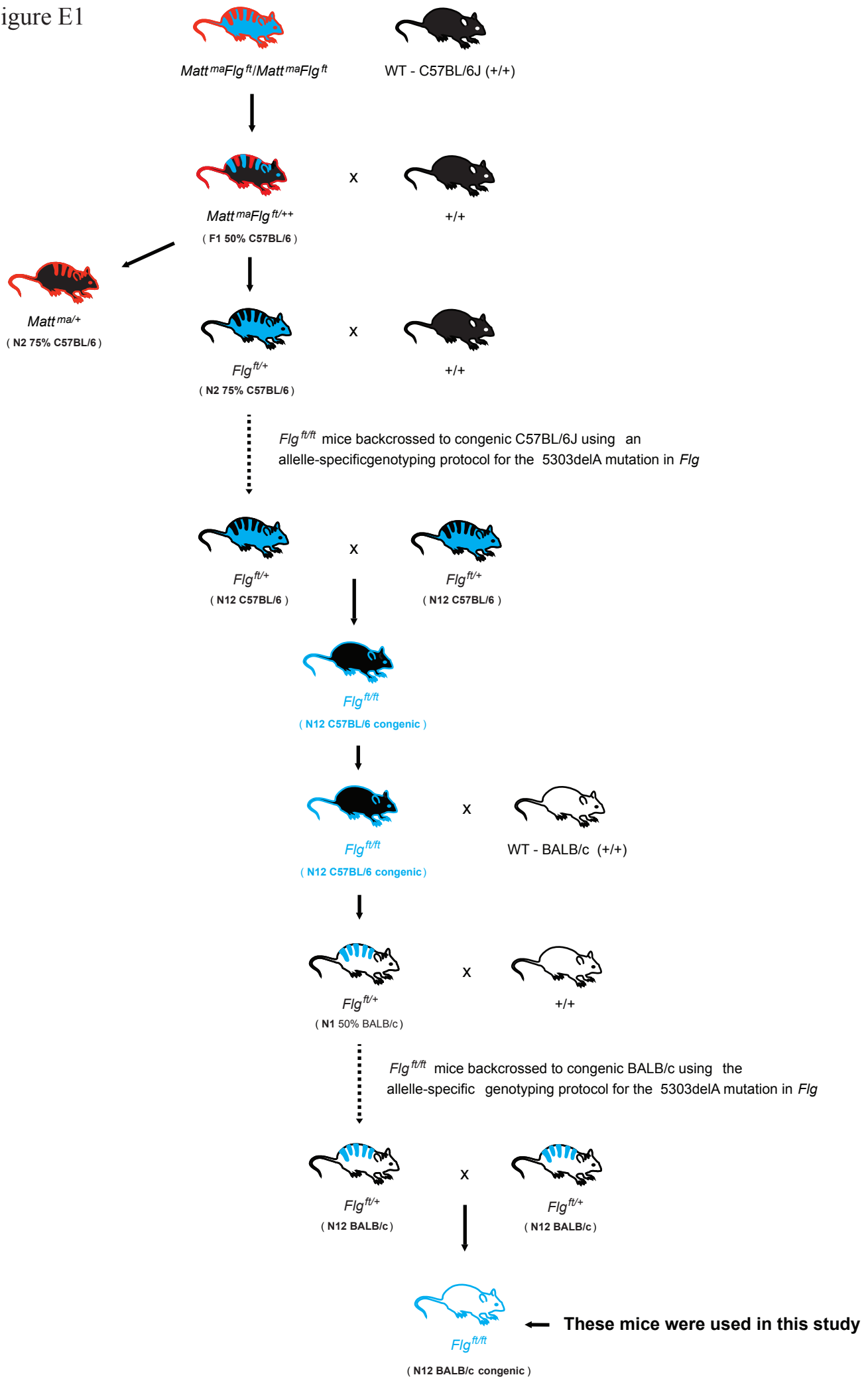

Supplement: Fig E1 [file mmc3.pdf]

Figure E2

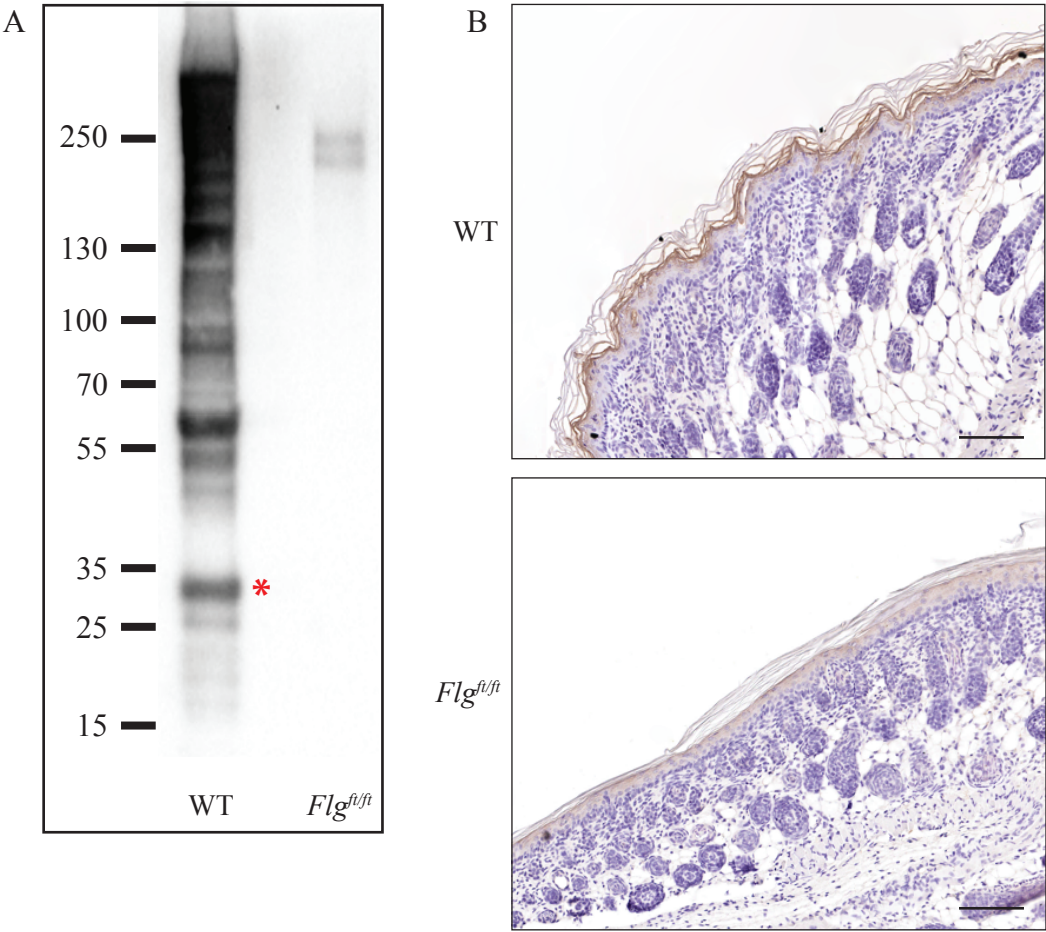

Supplement: Fig E2 [file mmc4.pdf]

Figure E3

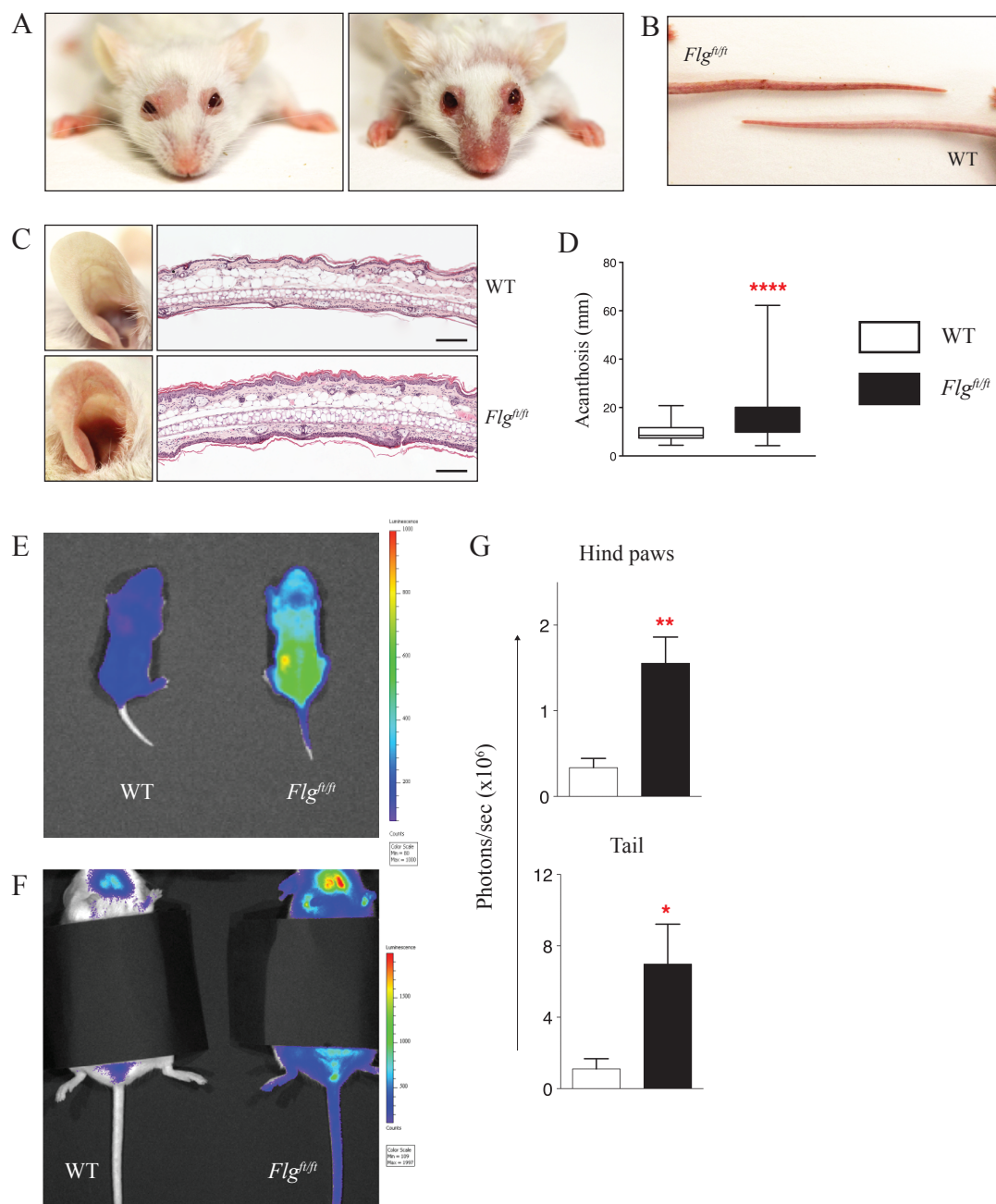

Supplement: Fig E3 [file mmc5.pdf]

Figure E4

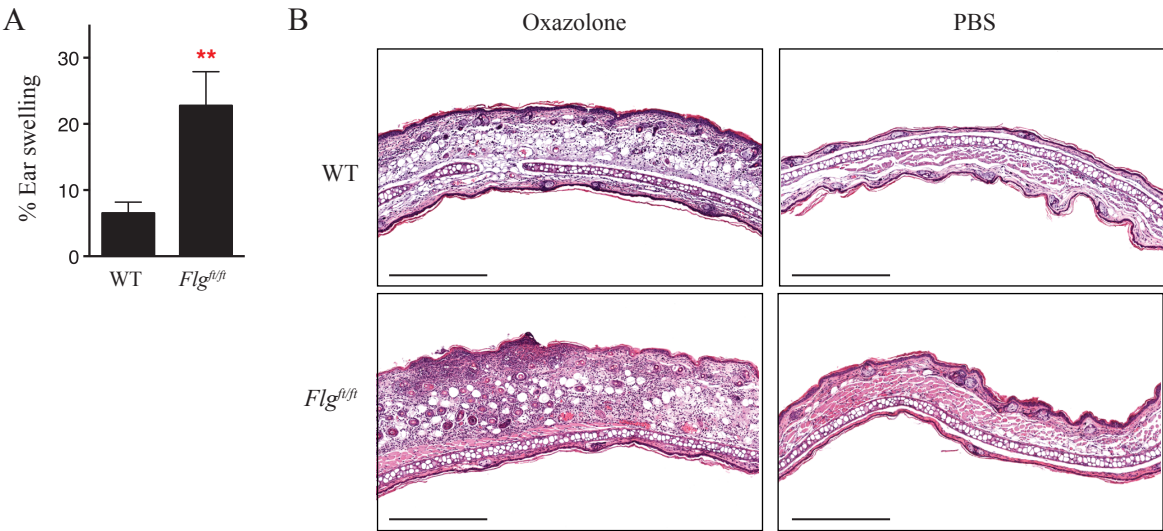

Supplement: Fig E4 [file mmc6.pdf]

Figure E5

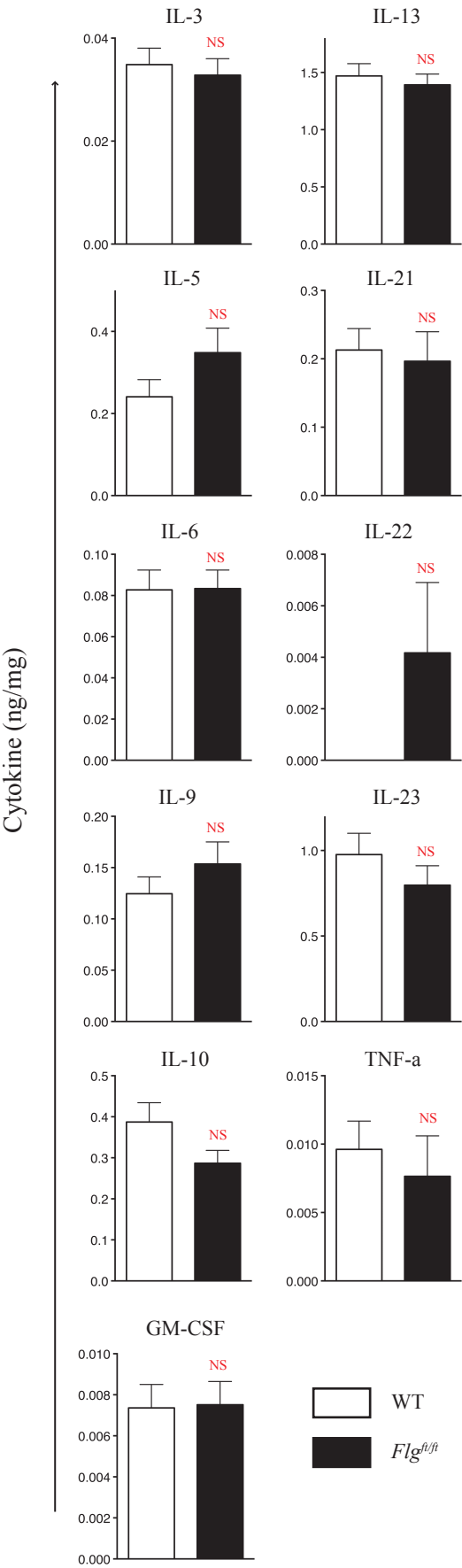

Supplement: Fig E5 [file mmc7.pdf]

Figure E6

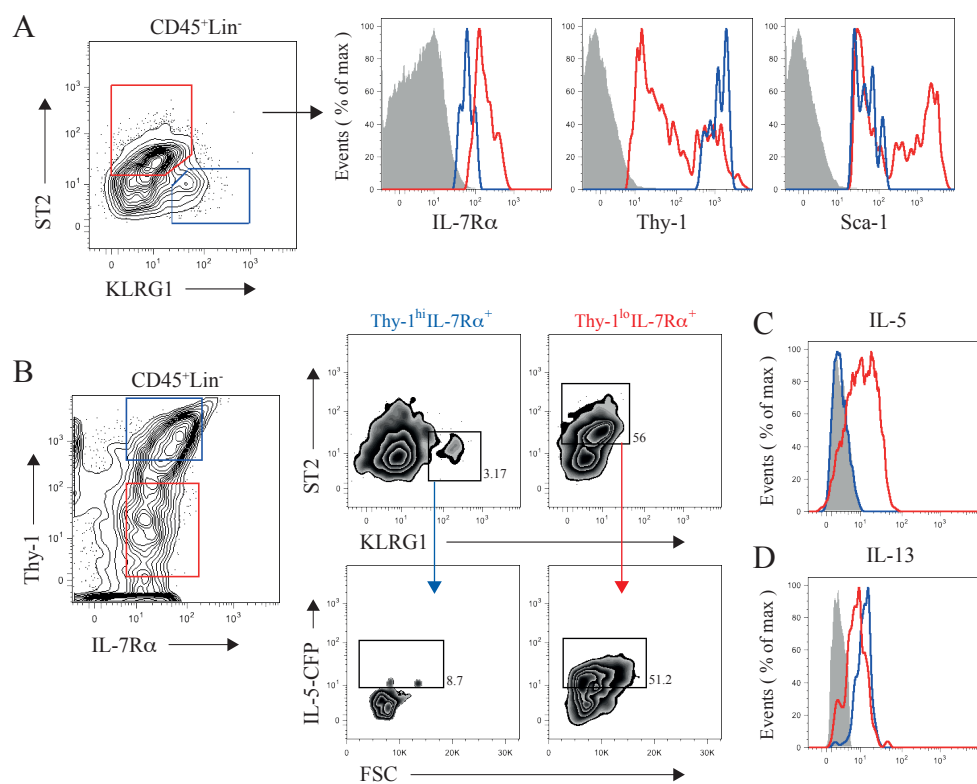

Supplement: Fig E6 [file mmc8.pdf]

Figure E7

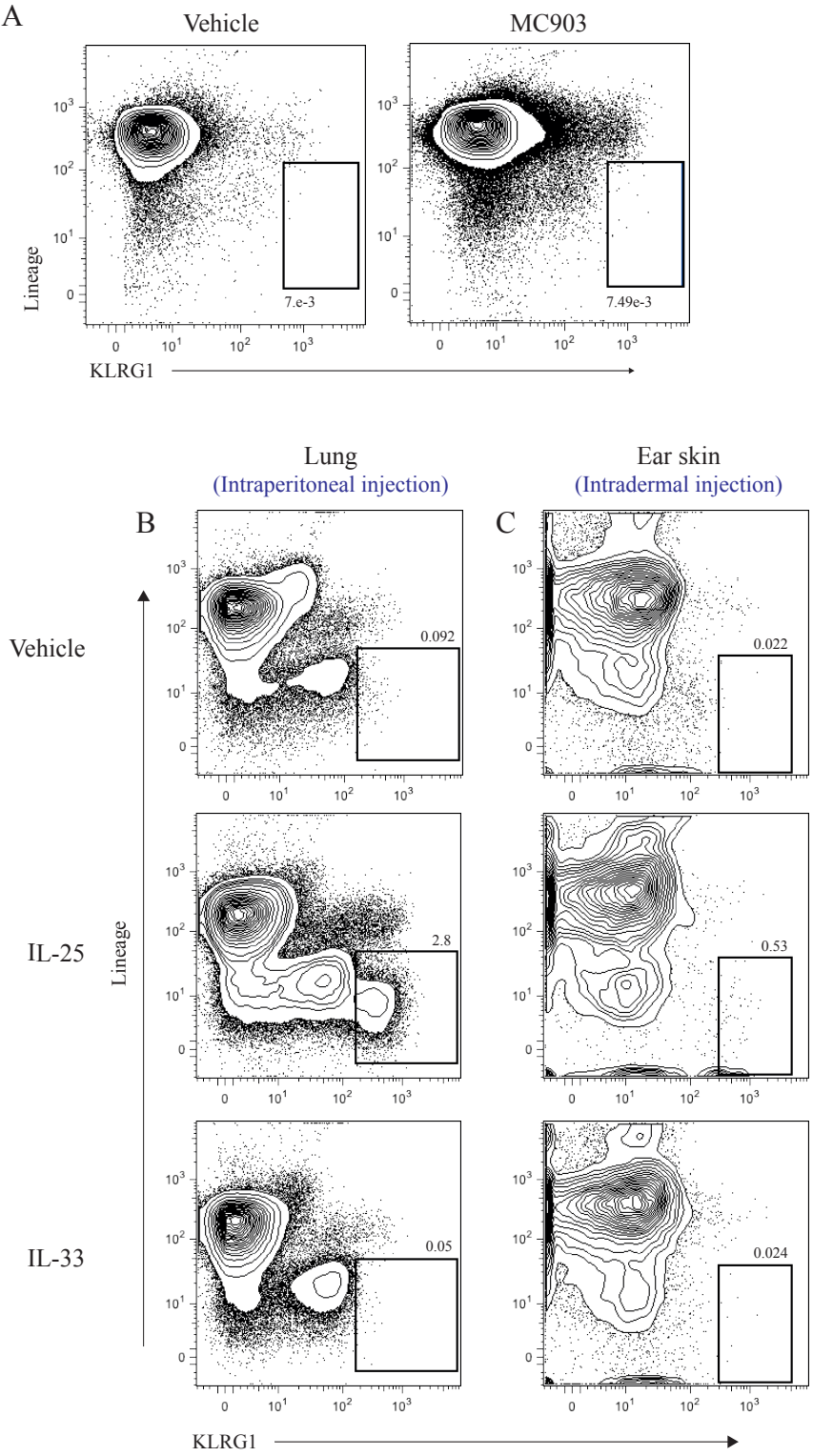

Supplement: Fig E7 [file mmc9.pdf]

Figure E8

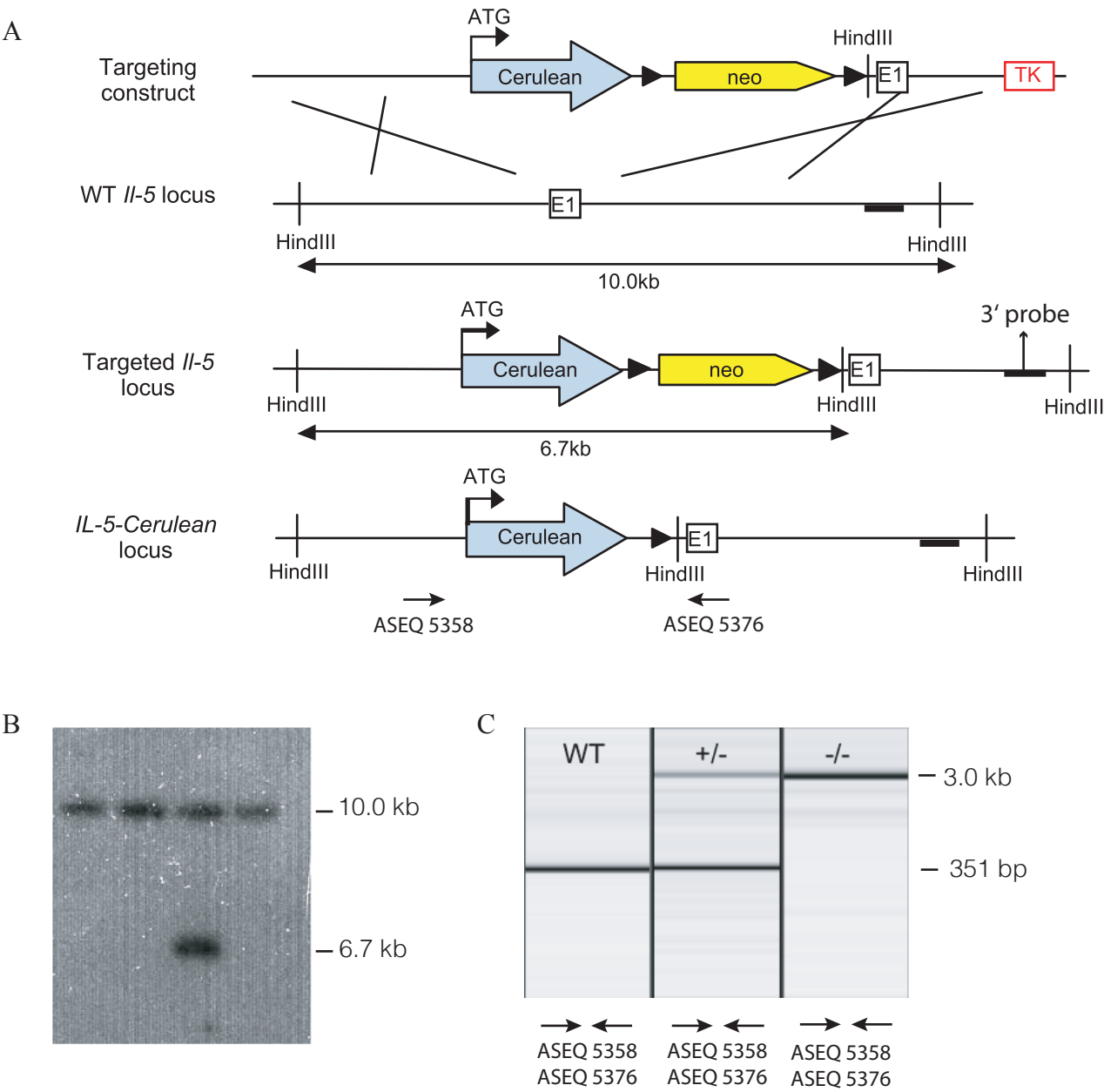

Supplement: Fig E8 [file mmc10.pdf]

Figure E9

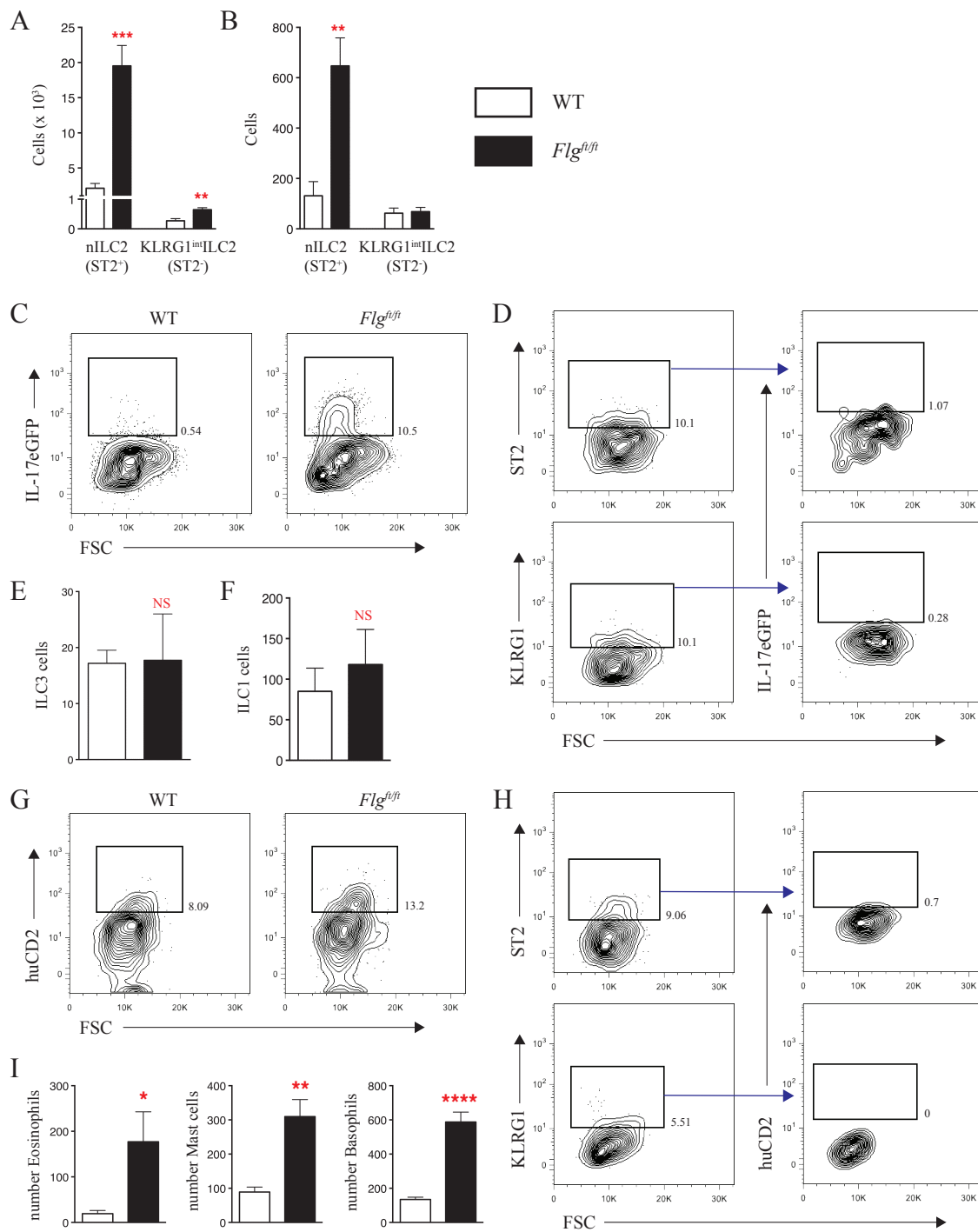

Supplement: Fig E9 [file mmc11.pdf]

Figure E10

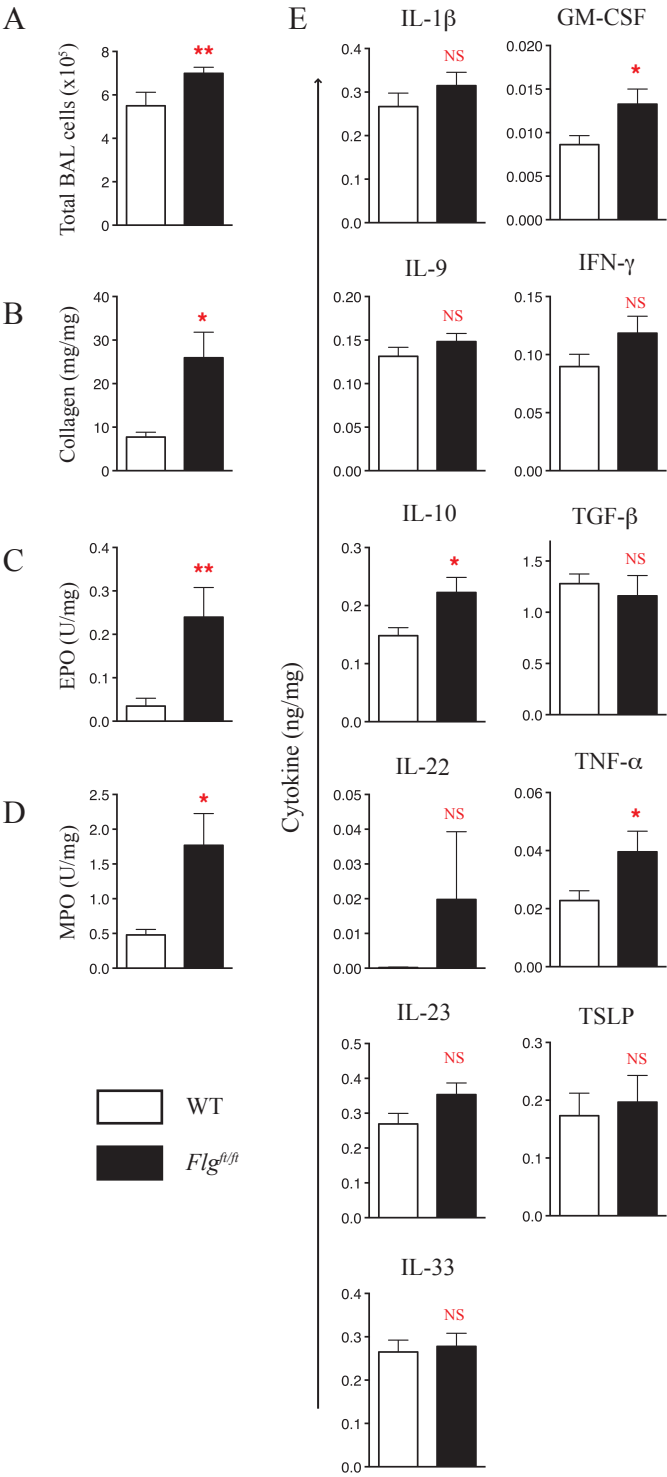

Supplement: Fig E10 [file mmc12.pdf]

Figure E11

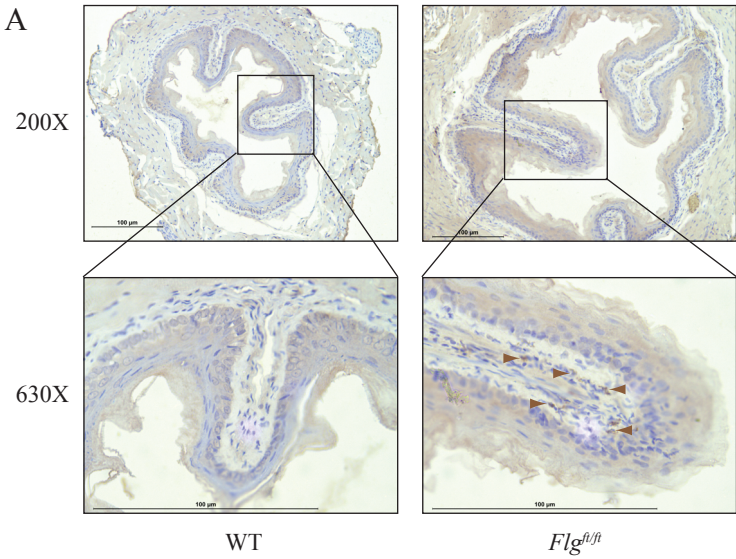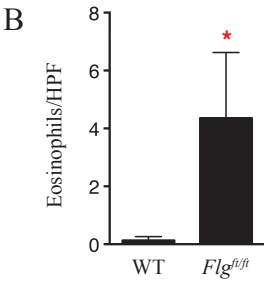

Supplement: Fig E11 [file mmc13.pdf]

Figure E13

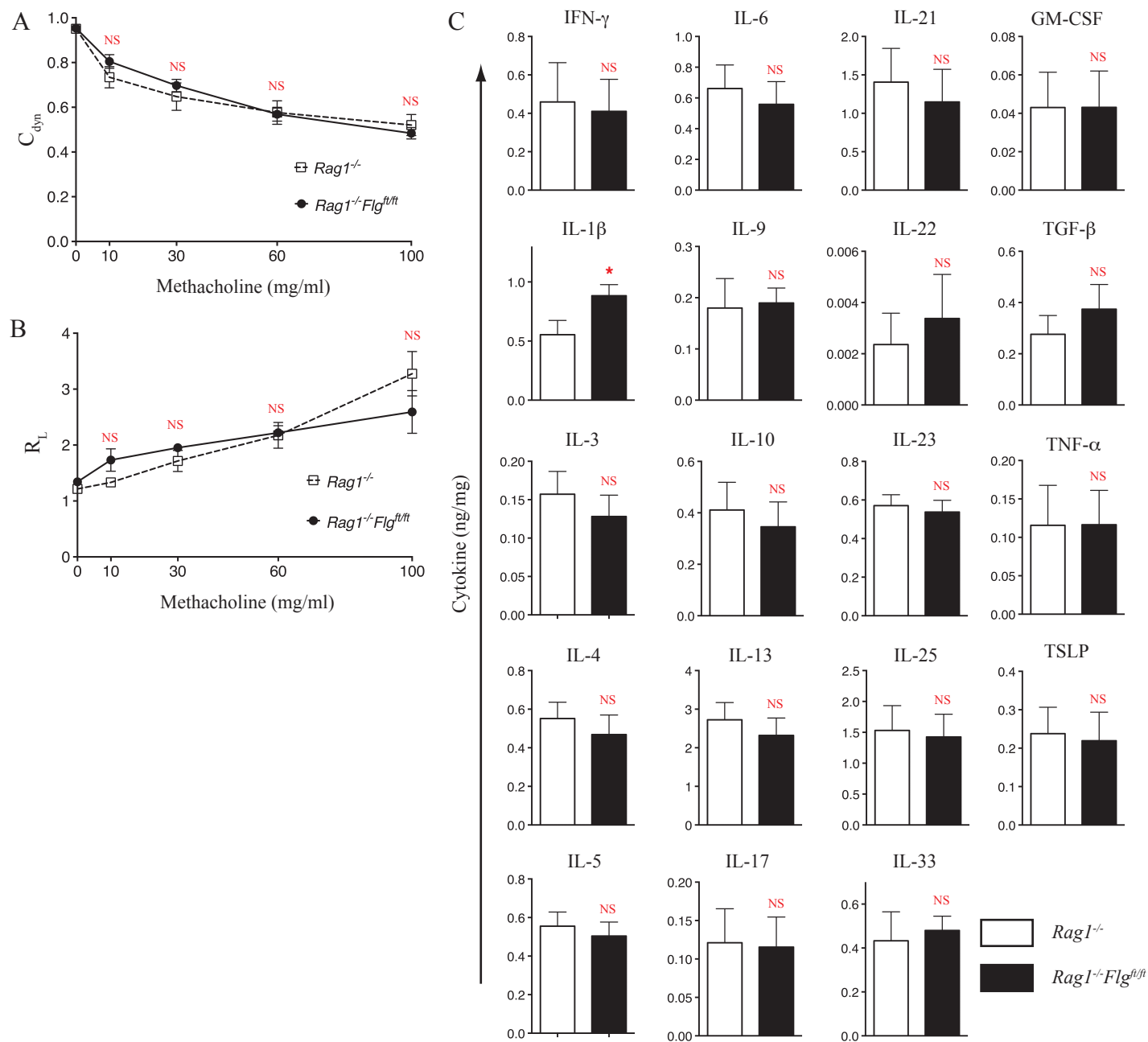

Supplement: Fig E13 [file mmc15.pdf]

Figure E14

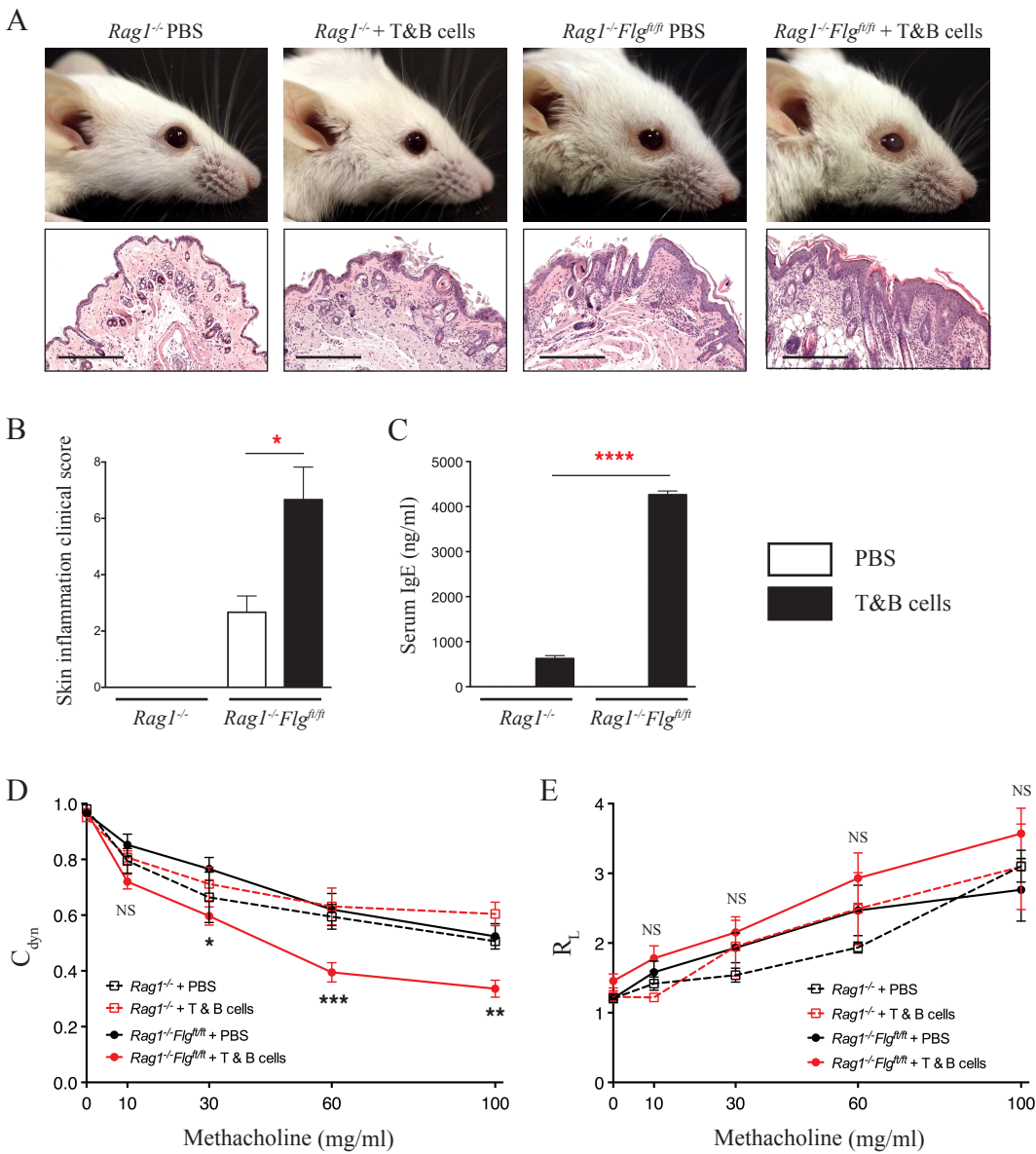

Supplement: Fig E14 [file mmc16.pdf]
