# Supplementary material for: Spontaneous atopic dermatitis is mediated by innate immunity, with the secondary lung inflammation of the atopic march requiring adaptive immunity
Source: J Allergy Clin Immunol. 2016 Feb;137(2):482–91. doi: 10.1016/j.jaci.2015.06.045 (PMC4735016; doi:10.1016/j.jaci.2015.06.045)
Supplement: Fig E12 [file mmc14.pdf]

Figure E12

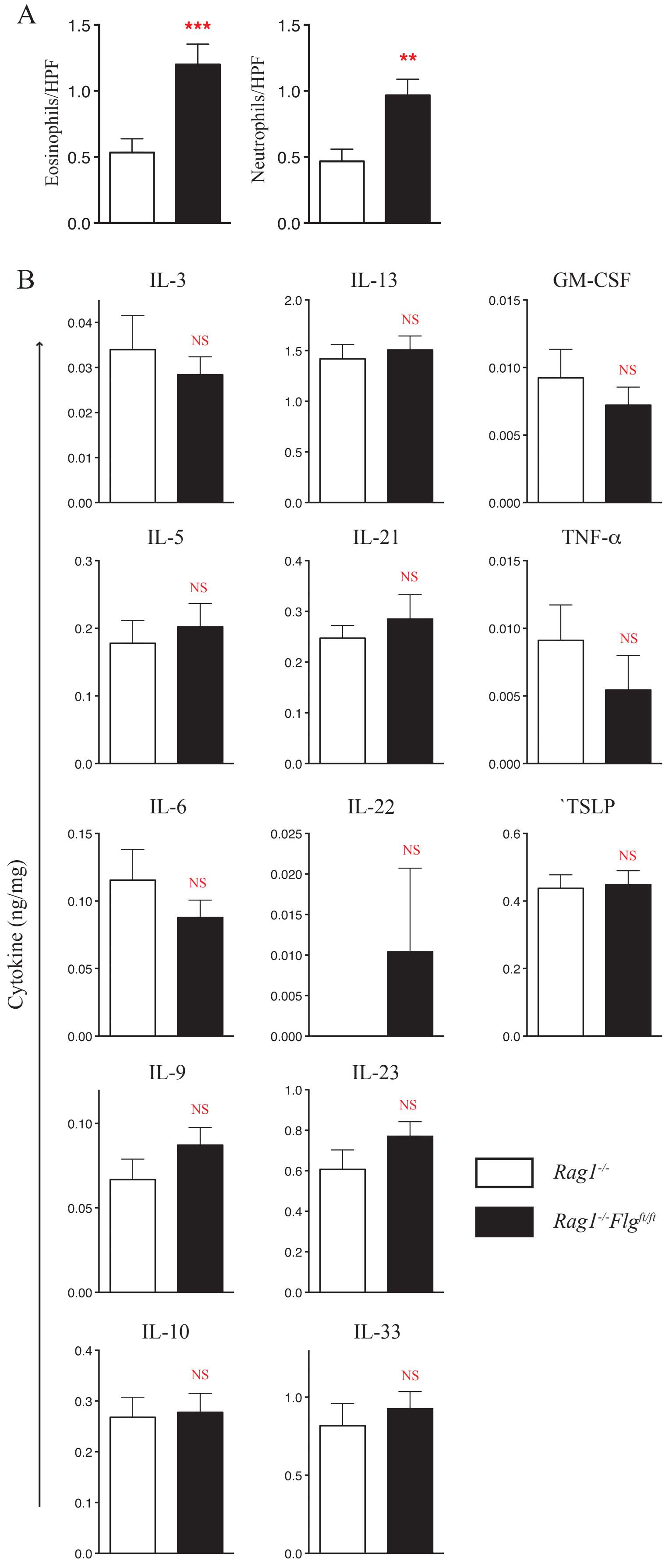

A
Eosinophils/HPF
Neutrophils/HPF
1.5
1.0
0.5
0.0
***
**
B
IL-3
IL-13
GM-CSF
IL-5
IL-21
TNF-α
IL-6
IL-22
`TSLP
IL-9
IL-23
IL-10
IL-33
NS
Cytokine (ng/mg)
Rag1-/-
Rag1-/-Flgft/ft
